# Author Correction: Insights into a dual function amide oxidase/macrocyclase from lankacidin biosynthesis

**DOI:** 10.1038/s41467-019-08571-w

**Published:** 2019-01-29

**Authors:** Jonathan Dorival, Fanny Risser, Christophe Jacob, Sabrina Collin, Gerald Dräger, Cédric Paris, Benjamin Chagot, Andreas Kirschning, Arnaud Gruez, Kira J. Weissman

**Affiliations:** 1 UMR 7365, Ingénierie Moléculaire et Physiopathologie Articulaire (IMoPA), CNRS-Université de Lorraine, Biopôle de l’Université de Lorraine, Campus Biologie Santé, 9 Avenue de la Forêt de Haye, BP 20199, 54505 Vandœuvre-lès-Nancy Cedex, France; 2 Institut für Organische Chemie, Leibniz Universität Hannover, Schneiderberg 1B, Hannover, 30167 Germany; 3 Laboratoire d’Ingénierie des Biomolécules, Ecole Nationale Supérieure d’Agronomie et des Industries Alimentaires (ENSAIA), Université de Lorraine, 2 Avenue de la Fôret de Haye, BP 172, 54518 Vandœuvre-lès-Nancy Cedex, France; 4 Present Address: Sorbonne Universités, UPMC Univ. Paris 06, CNRS, UMR 8227, Integrative Biology of Marine Models, Station Biologique de Roscoff, CS 90074 Roscoff, Bretagne France

Correction to: *Nature Communications*; 10.1038/s41467-018-06323-w; published online 28 September 2018

In the original version of this Article, the final concentration of riboflavin in the supplemented LB medium for recombinant LkcE expression was incorrectly stated as 1 g L^−1^ (this was the concentration of the stock solution) and should have read 10–50 mg L^−1^. This error has been corrected in both the PDF and HTML versions of the Article.

