# Author Correction: Insights into a dual function amide oxidase/macrocyclase from lankacidin biosynthesis

**DOI:** 10.1038/s41467-020-14473-z

**Published:** 2020-01-29

**Authors:** Jonathan Dorival, Fanny Risser, Christophe Jacob, Sabrina Collin, Gerald Dräger, Cédric Paris, Benjamin Chagot, Andreas Kirschning, Arnaud Gruez, Kira J. Weissman

**Affiliations:** 1 UMR 7365, Ingénierie Moléculaire et Physiopathologie Articulaire (IMoPA), CNRS-Université de Lorraine, Biopôle de l’Université de Lorraine, Campus Biologie Santé, 9 Avenue de la Forêt de Haye, BP 20199, 54505 Vandoeuvre-lès-Nancy Cedex, France; 2 Institut für Organische Chemie, Leibniz Universität Hannover, Schneiderberg 1B, Hannover, 30167 Germany; 3 Laboratoire d’Ingénierie des Biomolécules, Ecole Nationale Supérieure d’Agronomie et des Industries Alimentaires (ENSAIA), Université de Lorraine, 2 Avenue de la Fôret de Haye, BP 172, 54518 Vandoeuvre-lès-Nancy Cedex, France; 4 Present Address: Sorbonne Universités, UPMC Univ. Paris 06, CNRS, UMR 8227, Integrative Biology of Marine Models, Station Biologique de Roscoff, CS 90074 Roscoff, Bretagne France

**Keywords:** Enzyme mechanisms, X-ray crystallography, Biosynthesis

Correction to: *Nature Communications* 10.1038/s41467-018-06323-w, published online 28 September 2018

The original version of this Article contained errors in the reference numbering within the text. This has been corrected in both the PDF and HTML versions of the Article.

