# Supplementary material for: Insights into a dual function amide oxidase/macrocyclase from lankacidin biosynthesis
Source: Nat Commun. 2018 Sep 28;9:3998. doi: 10.1038/s41467-018-06323-w (PMC6162330; doi:10.1038/s41467-018-06323-w)
Supplement: Supplementary file 1 — Supplementary Information [file 41467_2018_6323_MOESM1_ESM.pdf]

Supplementary Information

Insights into a dual function amide oxidase/macrocyclase from lankacidin biosynthesis

Dorival, *et al.*

Supplementary Figures

a

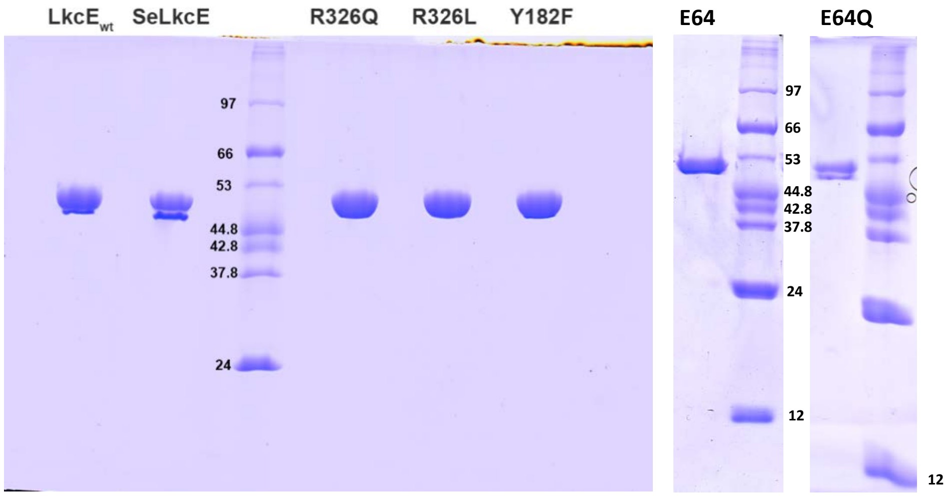

b

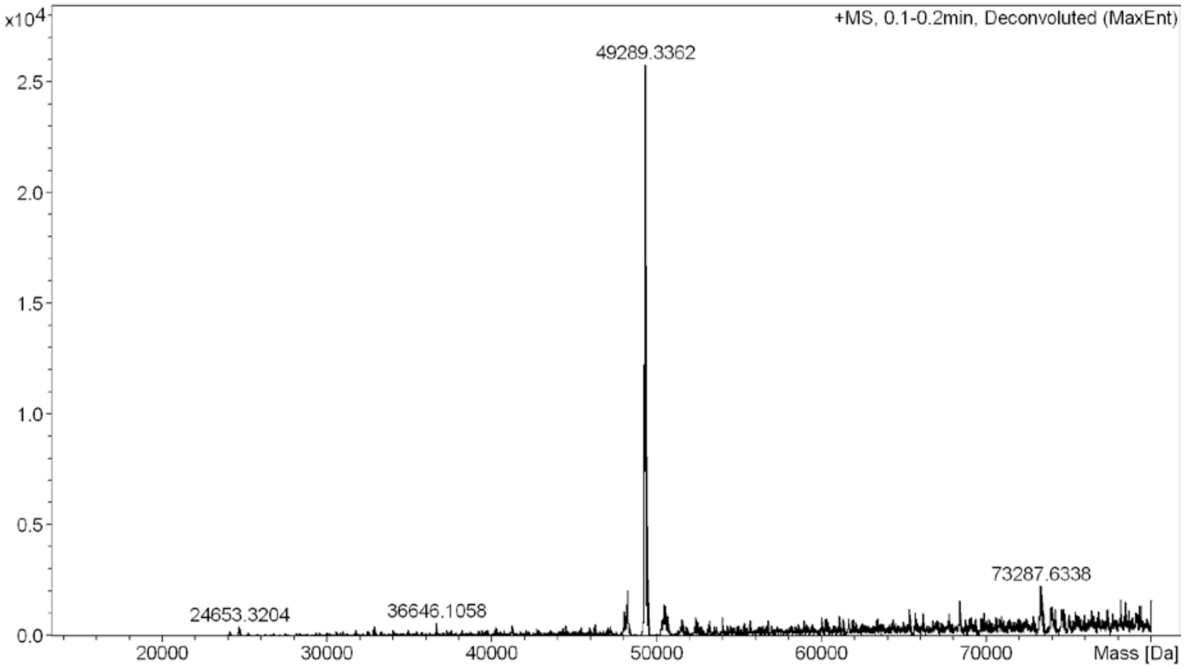

c

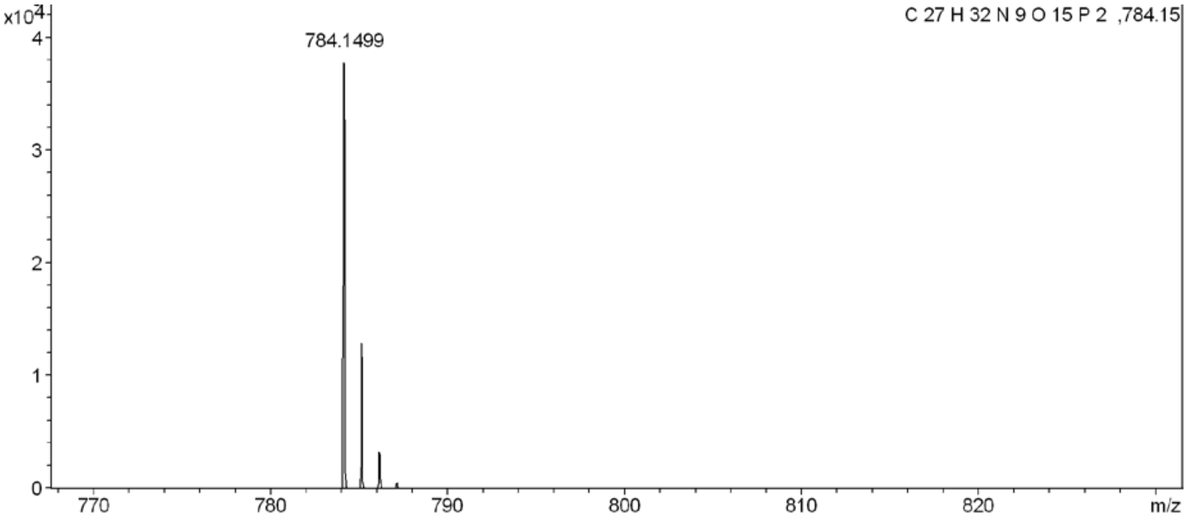

Supplementary Figure 1 │ Characterization of recombinant LkcE. (**a**) SDS-PAGE (12.5%) gels of purified LkcE (seleniated and non) and its 5 mutants. The molecular weights of the markers are indicated. (**b**) Deconvoluted mass spectrum of non-seleniated LkcE, giving a mass of 49,289.3 Da (vs. the theoretical mass of 49,289.4 Da). (**c**) Analysis by mass spectrometry of the liberated cofactor. The measured mass (m/z = 784.15 $[M–H]^-$) corresponds to that of FAD (785.5 Da).

a

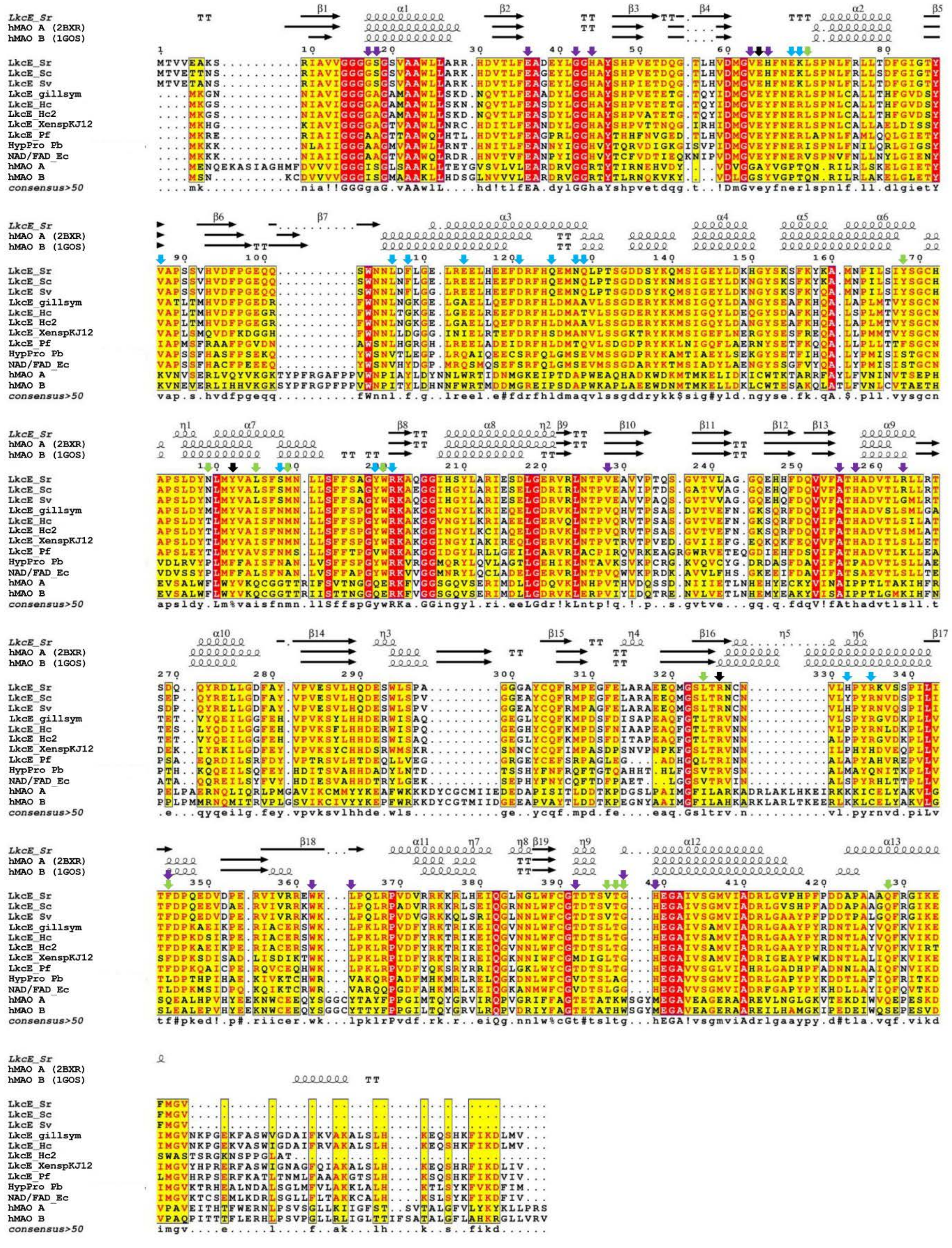

LkcE_Sr
hMAO A (2BXR)
hMAO B (1GOS)
LkcE_Sr
LkcE_Sc
LkcE_Sv
LkcE_gillsym
LkcE_Hc
LkcE_Hc2
LkcE_XenspKJ12
LkcE_Pf
HypPro_Pb
NAD/FAD_Ec
hMAO A
hMAO B
consensus>50

b

Analysis for the genomic context of the LkcE homologs

LkcE homolog (designation in (**a**))	NCBI gene sequence ID	Genomic context (translated protein products)
S. rochei var. *volubilis* (LkcE_Sr)	Not deposited, but differs by 1 amino acid (P424L) from WP_011113095 (ref. 3) and has the same genomic context	PqqB<br>PqqE<br>PqqD<br>PqqC<br>PqqA<br>ABC transporter<br>ABC transporter<br>Isochorismatase<br>LkcG<br>LkcF<br>**LkcE**<br>LkcD<br>LkcC<br>LkcB<br>LkcA<br>MaoC-like dehydrogenase<br>Efflux transporter<br>PCP<br>PKS FkbH-like<br>PQQ-dependent dehydrogenase
S. griseofuscus	WP_051850692	Prx<br>Anti-sigma factor<br>PKS protein (AT-KR-ACP-TE)<br>PQQ-dependent DH<br>LkcA<br>LkcB homolog? (KS-DH?)<br>**LkcE**<br>(2 proteins which have been removed from the database)<br>MerR family transcriptional regulator<br>Hypothetical protein
S. cellostaticus (LkcE_Sc)	KUM93544	PQQ-dependent dehydrogenase<br>PKS FkbH-like<br>Carrier protein homolog<br>SDR homolog<br>LkcA<br>LkcB<br>LkcC<br>LkcD<br>**LkcE**<br>LkcF<br>LkcG<br>Nicotinamidase<br>ABC transporter<br>ABC transporter<br>PqqC<br>PqqD<br>PqqE<br>PqqB
S. yokosukanensis (LkcE_Sy)	WP_067137025	LkcG<br>LkcF<br>**LkcE**<br>LkcD<br>Nicotinamidase[a]
Solemya velum gill symbiont (LkcE_gillsym)	WP_078462125	LkcG<br>ACP-KS?<br>KS?<br>KS-KR-ACP-KS (portion of LkcF?)<br>KR-ACP (portion of LkcF?)<br>Nicotinamidase<br>**LkcE**<br>LkcD<br>LkcC

		LkcB<br>LkcA<br>Sfp-type phosphopantetheinyl transferase (PPTase)<br>ACP?
H. chejuensis<br>(LkcE_Hc)	CCG06111	LkcG<br>LkcF<br>Isochorismatase<br>**LkcE**<br>LkcD<br>LkcC<br>LkcB<br>LkcA<br>Transcriptional regulator
H. chejuensis<br>(LkcE_Hc2)	WP_011397301	Carrier protein homolog<br>MFS transporter<br>LkcG<br>LkcF<br>Nicotinamidase<br>**LkcE**<br>LkcD<br>LkcC<br>LkcB<br>LkcA
Xenorhabdus sp. KJ12.1<br>(LkcE_XenspKJ12)	WP_099111721	MFS transporter<br>LkcG<br>LkcF<br>Nicotinamidase<br>**LkcE**<br>LkcD<br>LkcC<br>LkcB<br>LkcA<br>Carrier protein homolog
Pseudomonas fluorescens<br>(LkcE_Pf)	WP_096820043	ABC transporter<br>Sfp-type PPTase<br>LkcG<br>LkcF<br>Nicotinamidase<br>**LkcE**<br>LkcD<br>LkcC<br>LkcB<br>LkcA<br>MFS transporter
Pectobacterium betavasculorum<br>(HypPro_Pb)	WP_072012528	Hypothetical protein<br>Record removed<br>**LkcE homolog**<br>Hypothetical protein<br>Paraquat-inducible protein A<br>Hypothetical protein
E. coli<br>(NAD/FAD_Ec)	WP_096961323	PhzD<br>PhzE<br>PhzF<br>PhzG<br>AMP-dependent ligase<br>Discrete AT<br>A-PCP<br>Hypothetical protein<br>**LkcE homolog**<br>3-deoxy-7-phosphoheptulonate synthase<br>PPTase

[a]On either side of these genes, the NCBI numbers correspond to genes present in *S. celestaticus*.

Supplementary Figure 2 │ Analysis of LkcE homologs. (**a**) Multiple sequence alignment of LkcE with various homologs. The sequences include LkcE from *Streptomyces rochei* and its 9 closest homologs identified using BlastP[4], as well as human amine oxidases A and B, the closest amine oxidase homologs for which structures are available (PDB accession codes 2BXR and 1GOS, respectively) (the secondary structure elements present in each structure are indicated). The sequences include: LkcE *Steptomyces rochei* (Sr) (100% identity with WP_051850692 in *Streptomyces griseofuscus*, which lies in a partial lankacidin cluster (see part (**b**)); LkcE *Streptomyces cellostaticus* (Sc) (KUM93544): 91% I; 94% S, almost complete lankacidin cluster; LkcE *Streptomyces yokosukanensis* (Sy) (KUM94155): 91% I; 94% S, only partial cluster information available; LkcE (NAD/FAD-binding protein *Solemya velum* gill symbiont) (gillsym) (WP_078462125) 61% I; 79% S, partial lankacidin cluster; LkcE *Hahella chejuensis* (Hc) (CCG061111): 63% I; 79% S, lies in the chejuenolide cluster, which is responsible for biosynthesis of a close relative of lankacidin C[5]; LkcE *Hahella chejuensis* (Hc2) (WP_011397301): 61% I; 79% S, partial lankacidin/chejuenolide cluster; LkcE (NAD/FAD-binding protein *Xenorhabdus* species KJ12.1) (WP_099111721) 56% I; 71% S, partial lankacidin cluster; LkcE *Pseudomonas fluorescens* (Pf) (WP_096820043): 54% I; 66%S, partial lankacidin cluster; hypothetical protein *Pectobacterium betavasculorum* (HyPro_Pb) (WP_072012528): 46% I; 65% S; does not lie in a lankacidin cluster; NAD/FAD binding protein *Escherichia coli* (NAD/FAD_Ec) (WP_096961323): 46% I; 64% S, does not lie in a lankacidin cluster; Human amine oxidase A, 25% I; Human amine oxidase B, 25% I. Key to color-coding of residues indicated by arrows: purple = residues implicated directly in FAD binding; black = residues mutated in this study; green = residues observed to interact with bound LC-KA05; blue = residues contributing to LkcE homodimerization. (**b**) Genomic context supporting assignment of 7 *S. rochei* LkcE homologs (among the 9 in (**a**)) as lankacidin cyclases.

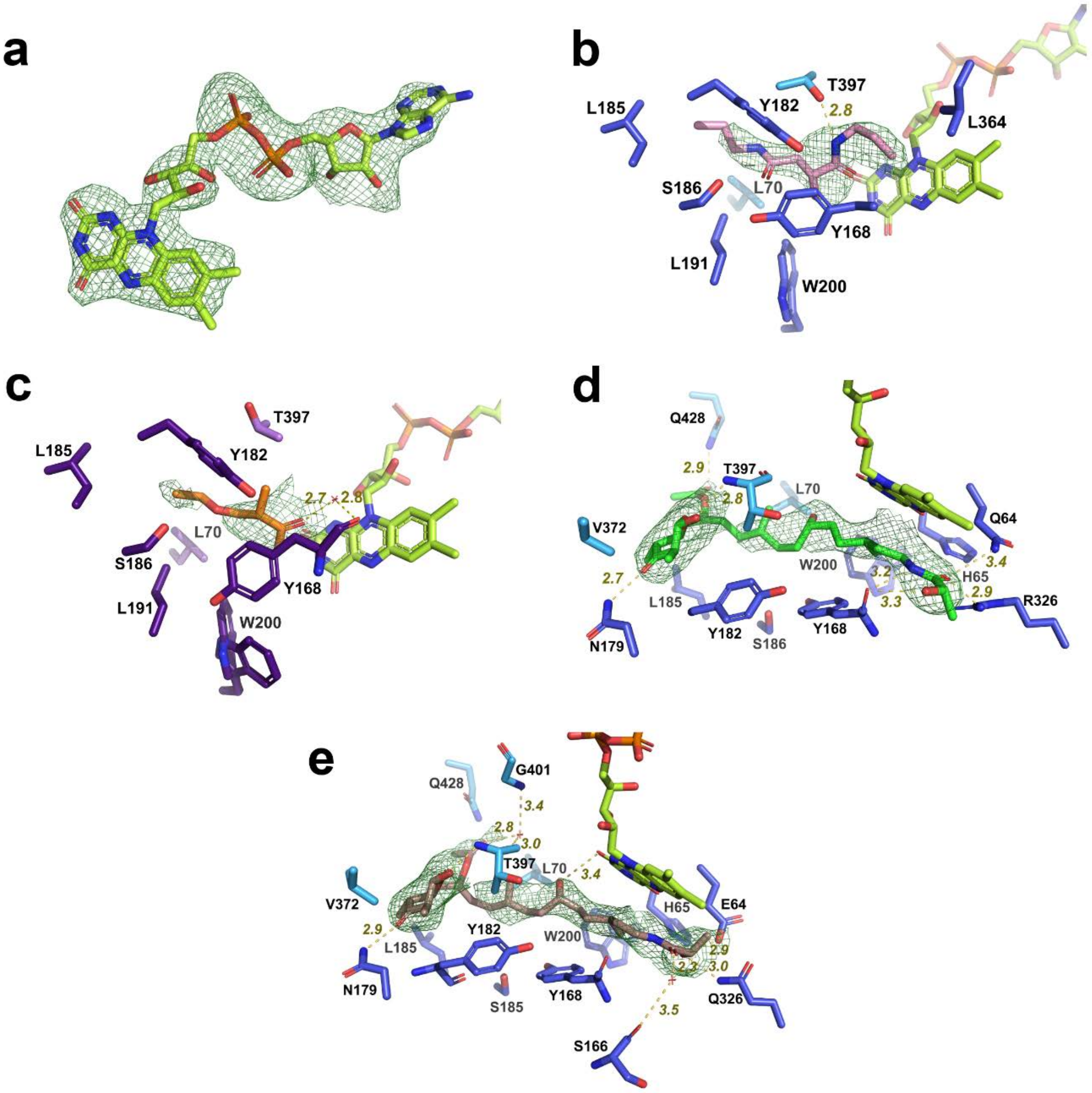

Supplementary Figure 3 │ Omit maps for FAD and ligands bound into LkcE. (**a**) Omit-map calculated for the FAD (yellow) in the structure of the wild type LkcE. The F_o–F_c map is contoured at 2.5σ. (**b**) Omit-map calculated for the structure of the wild type LkcE. The F_o–F_c electron density corresponding to bound DATD (pink) is contoured at 2.5σ. (**c**) View of the active site of wild type LkcE in the presence of bound EMAA (orange) with the omit F_o–F_c map contoured at 1.5σ. (**d**) Omit-map calculated for the structure of the LkcE E64Q mutant in the presence of the bound LC-KA05 (green). The F_o–F_c map contoured at 2.5σ indicates the position of the bound substrate. (**e**) View of the active site of R326Q mutant in the presence of bound LC-KA05 (brown) with the omit F_o–F_c map contoured at 2.5σ.

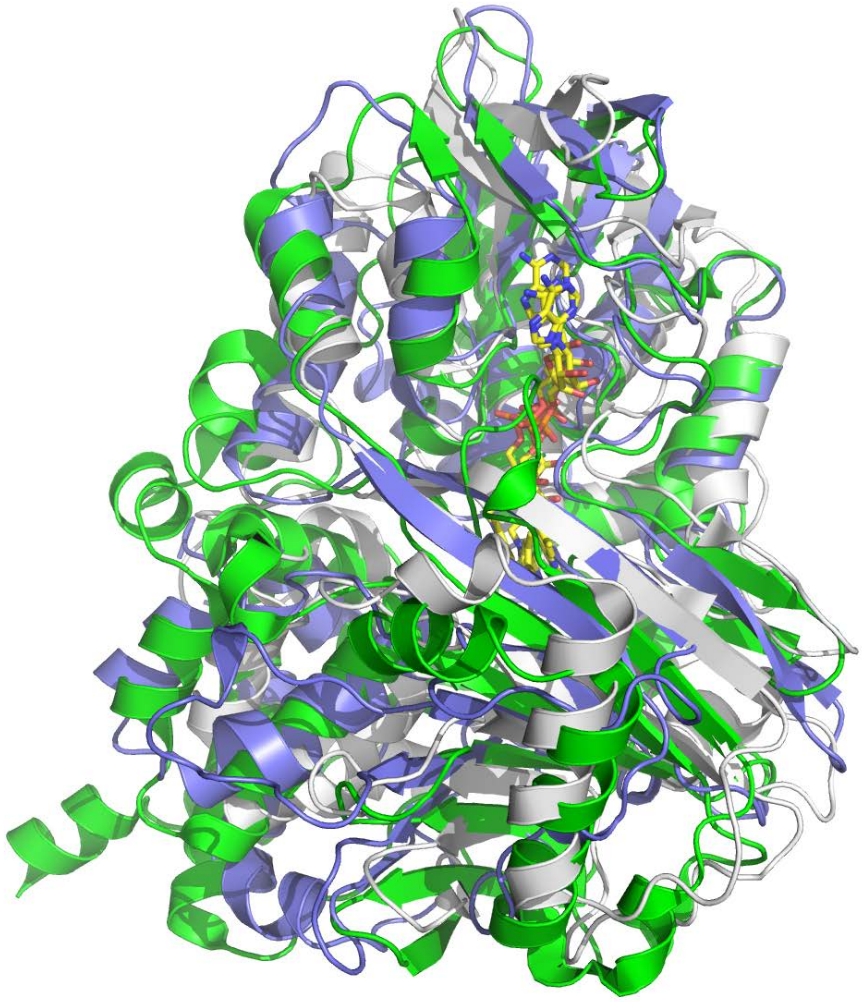

Supplementary Figure 4 │Superposition of a monomer of LkcE with the monomers of two structural homologs. The LkcE monomer is shown in violet, while those of 6HDNO (PDB 3NG7)[6] and hMAO B (PDB 1GOS)[7] are represented in grey and green, respectively. The FAD cofactor present in each structure is shown in stick form, with conventional atom coloring. This superposition illustrates in a qualitative manner that the cofactor binding domains are more highly conserved than the substrate-binding domains.

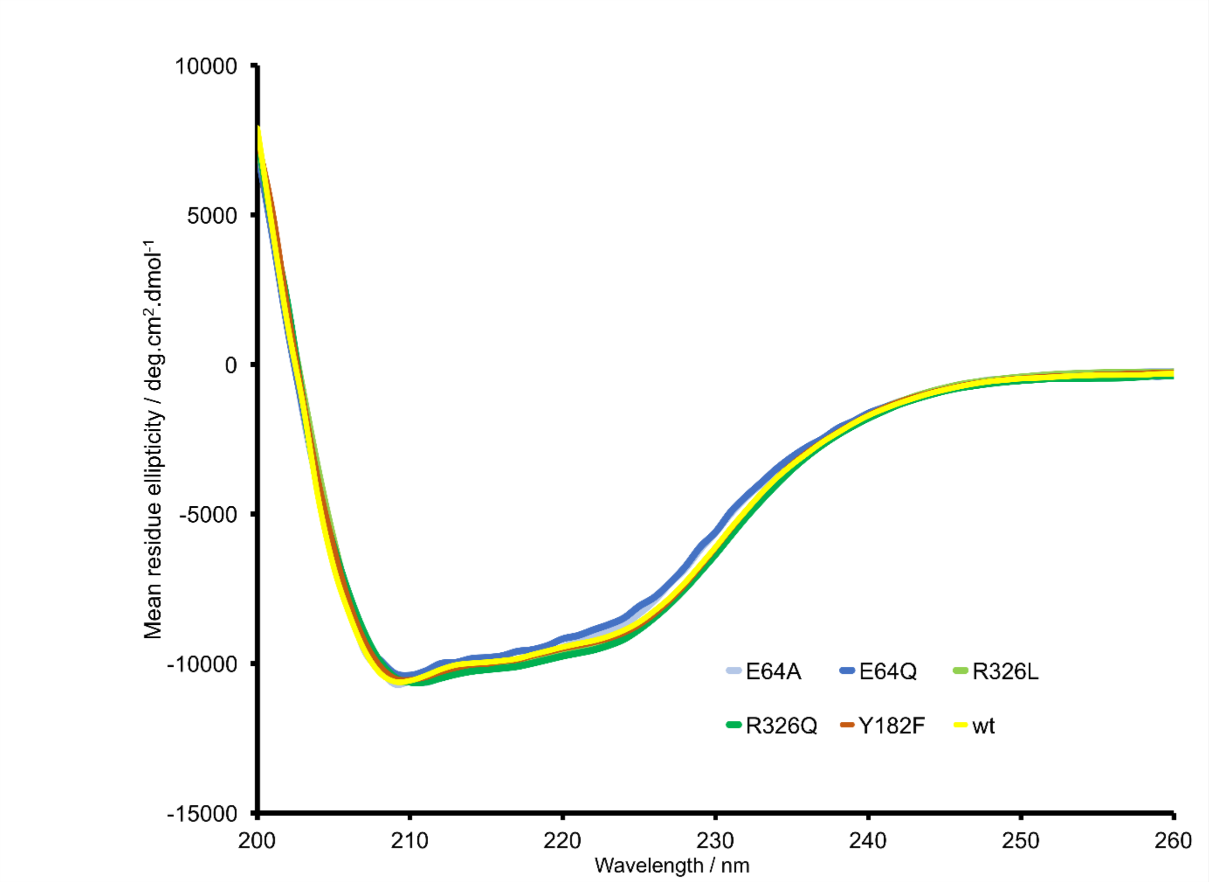

Supplementary Figure 5 │ Analysis by circular dichroism of LkcE wild type and its five active site mutants. As the spectra are essentially superimposable, this analysis showed that the mutations did not significantly alter the structure of the enzyme.

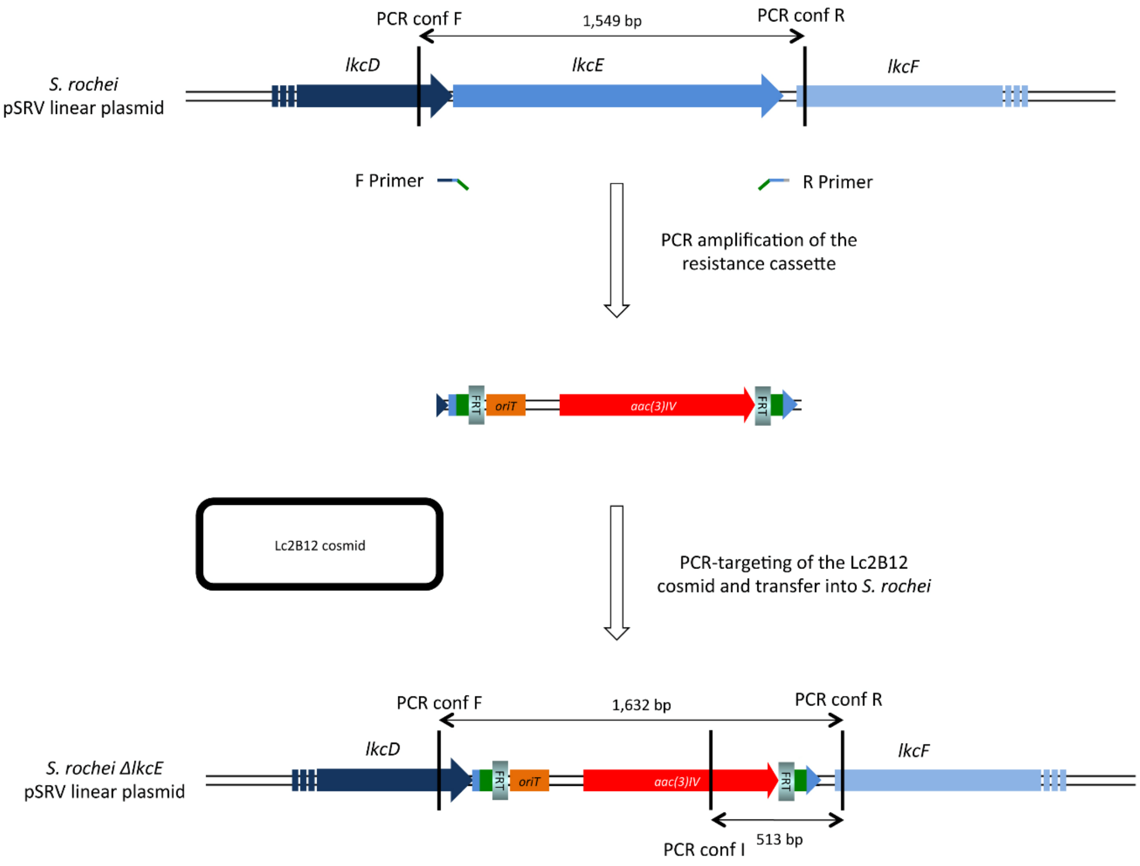

Supplementary Figure 6 │ Schematic illustration of the *lkcE* gene disruption in *Streptomyces rochei* by PCR-targeting[1]. The resistance cassette amplified from the pIJ773 plasmid using the F and R primers (**Supplementary Table 2**) was used to replace the *lkcE* gene in the cosmid Lc2B12 (Kan^R). The mutant cosmid was then transformed into *S. rochei* and clones (Kan^S Apr^R) were selected and analyzed by PCR (the binding locations of the primers use to confirm the deletion (PCR conf F, PCR conf R and PCR conf I (**Supplementary Table 2**)) are indicated).

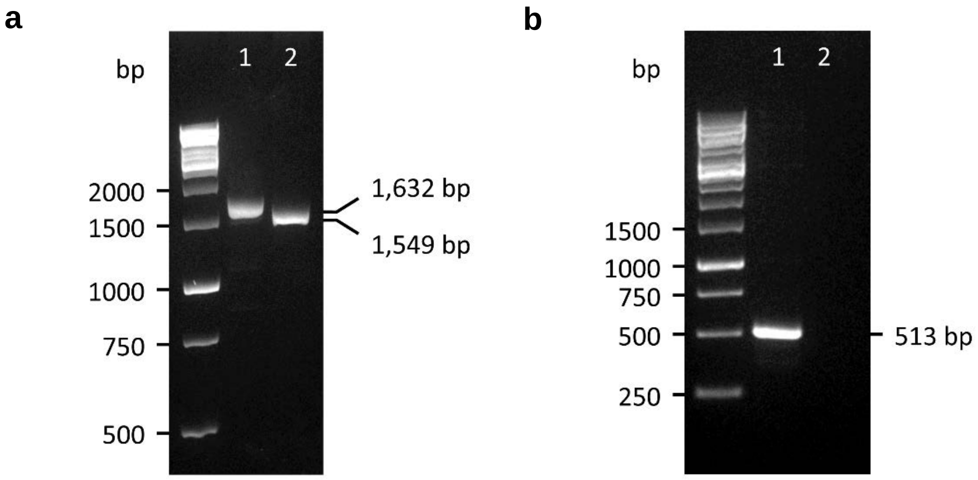

Supplementary Figure 7 │PCR analysis of the lankacidin biosynthetic gene cluster using genomic DNA from *S. rochei* Δ*lkcE* (lanes 1 in panels (**a**) and (**b**)) **and *S. rochei* WT** (lanes 2 in panels (**a**) and (**b**)) **strains.** (**a**) The product of amplification of the wild type *lkcE* locus using primers conf F/R has the expected size of 1549 bp (as seen in lane 2), while replacement of *lkcE* by the apramycin resistance cassette gives a product of 1632 bp (as seen in lane 1). (**b**) No product is expected with the wild type strain and primers conf I/R (as with lane 2), while when the apramycin resistance cassette is present, this primer pair yields a product of 513 bp (as in lane 1).

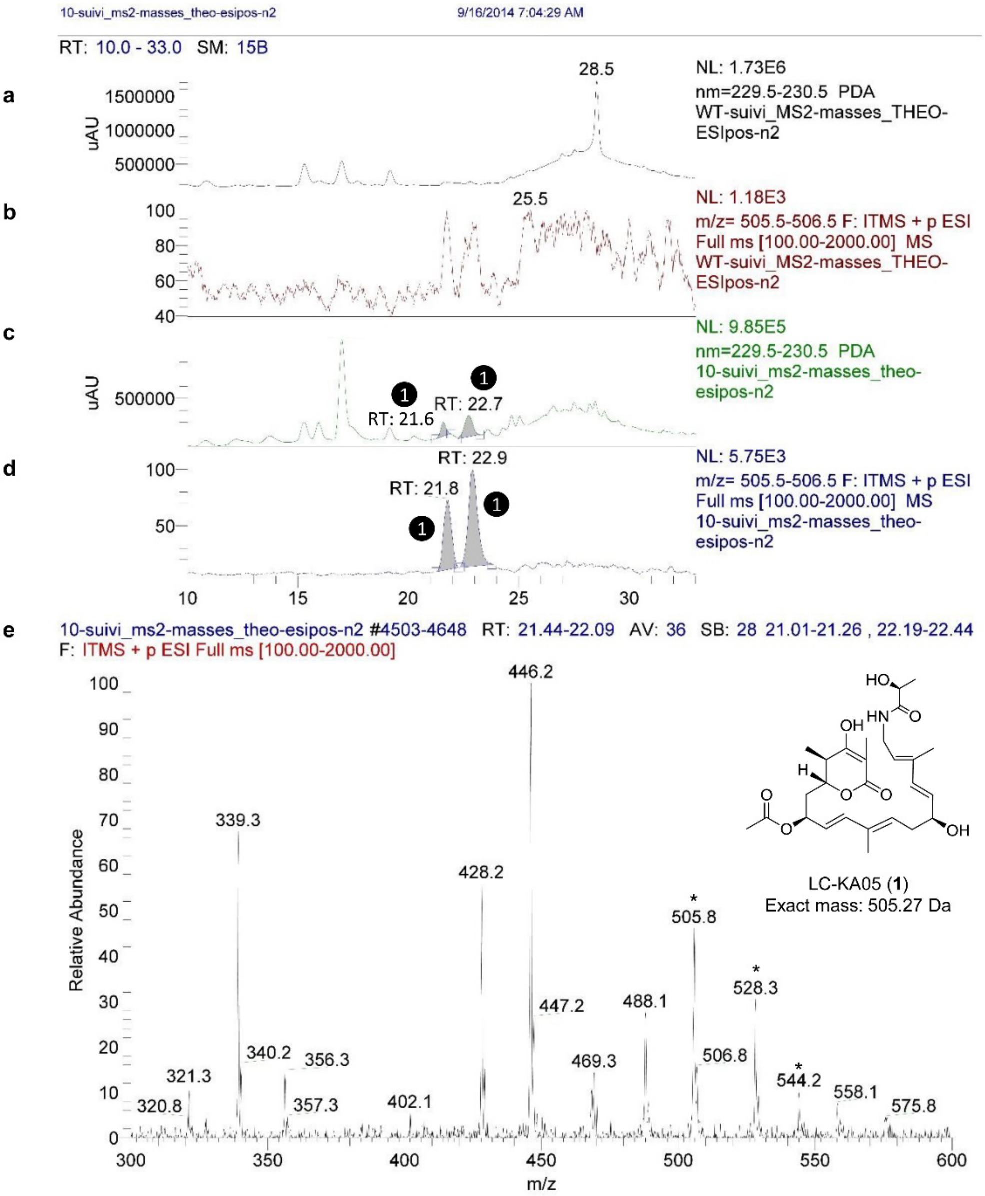

Supplementary Figure 8 │ Analysis by HPLC-MS of extracts of wild type *S. rochei* vs. the Δ*lkcE* inactivation mutant for LC-KA05 (1) and downstream products. *m/z* values obtained using the ion trap are reported to one significant figure, while those acquired in the Orbitrap mode are reported to two significant figures. (**a**)–(**i**) Analysis for the presence of LC-KA05 (**1**) (exact mass = 505.27 Da). (**a**) Trace of the absorbance at 230 nm of extracts of the wild type. (**b**) SIM of *m/z* = 506 (ESI positive) of extracts of the wild type. (**c**) Trace of the absorbance at 230 nm of extracts of the Δ*lkcE* inactivation mutant. The two peaks corresponding to **1** (as indicated), which are not observed in wild type extracts, are greyed. (**d**) SIM of *m/z* = 506 (ESI positive) of extracts of the Δ*lkcE* inactivation mutant. The two peaks corresponding to **1** are greyed. (**e**) Mass spectrum of the peak at RT = 21.8 min (which is representative of the second peak at 22.9 min), showing ions consistent with **1** ($[M+H]^+$ = 505.8, $[M+Na]^+$ = 528.3, $[M+K]^+$ = 544.2, starred).

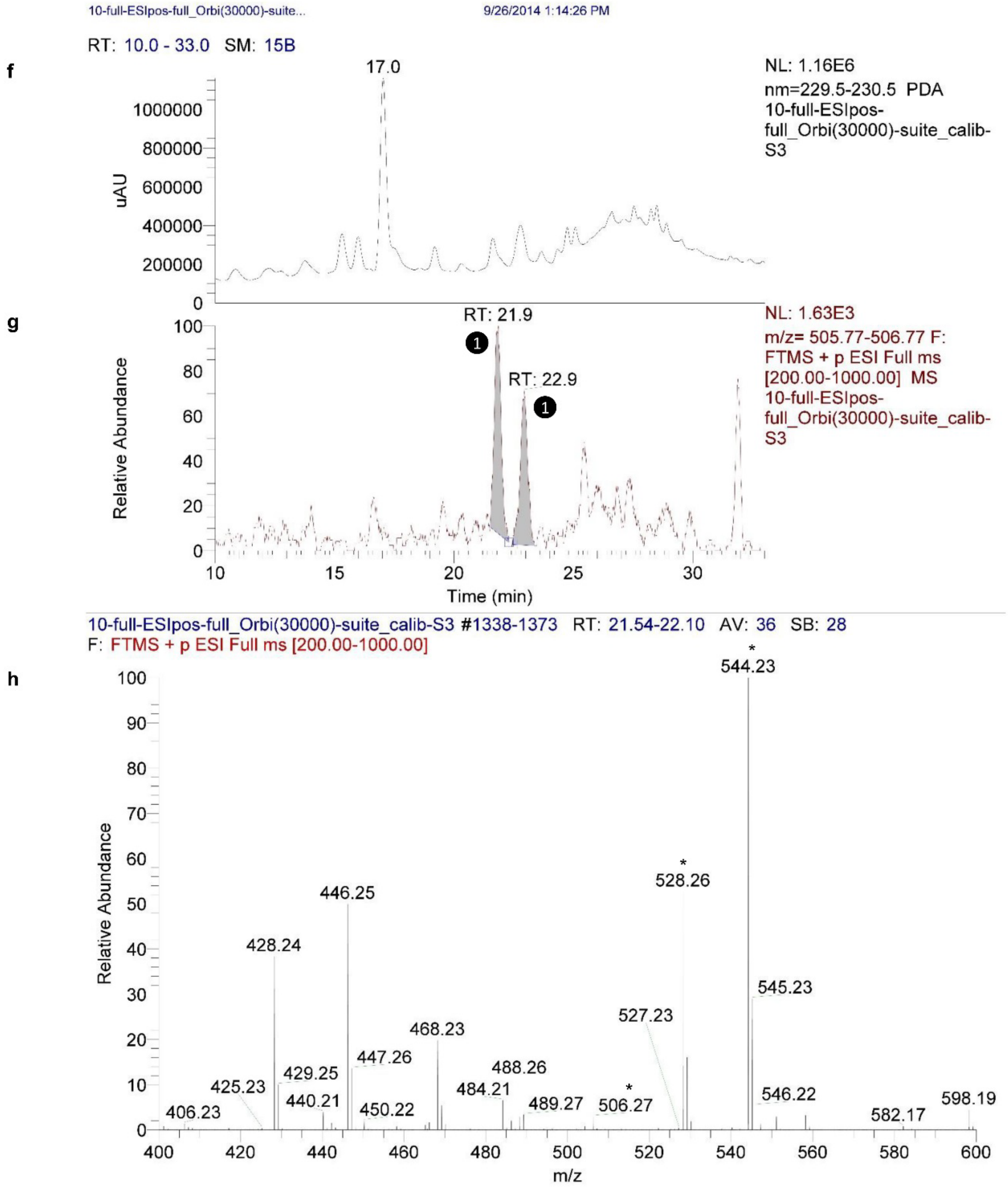

Supplementary Figure 8 │ Analysis by HPLC-MS of extracts of wild type *S. rochei* vs. the Δ*lkcE* inactivation mutant for LC-KA05 (1**) and downstream products, cont.** Nonetheless, as the *m/z* ratio of 505.8 was lower than expected for LC-KA05 (i.e. *m/z* = 506.3 Da), we reanalyzed the extracts at high resolution (Orbitrap mode (panels (**f**)–(**h**)). (**f**) Trace of the absorbance at 230 nm of extracts of the Δ*lkcE* inactivation mutant. (**g**) SIM of *m/z* = 506.27 of the Δ*lkcE* inactivation mutant. This analysis yielded peaks of the same retention time as in (**d**). (**h**) Mass spectrum of the peak at RT = 21.9 min, confirming the expected exact mass for LC-KA05 (**1**) (starred – $[M+H]^+$, $[M+Na]^+$ and $[M+K]^+$)).

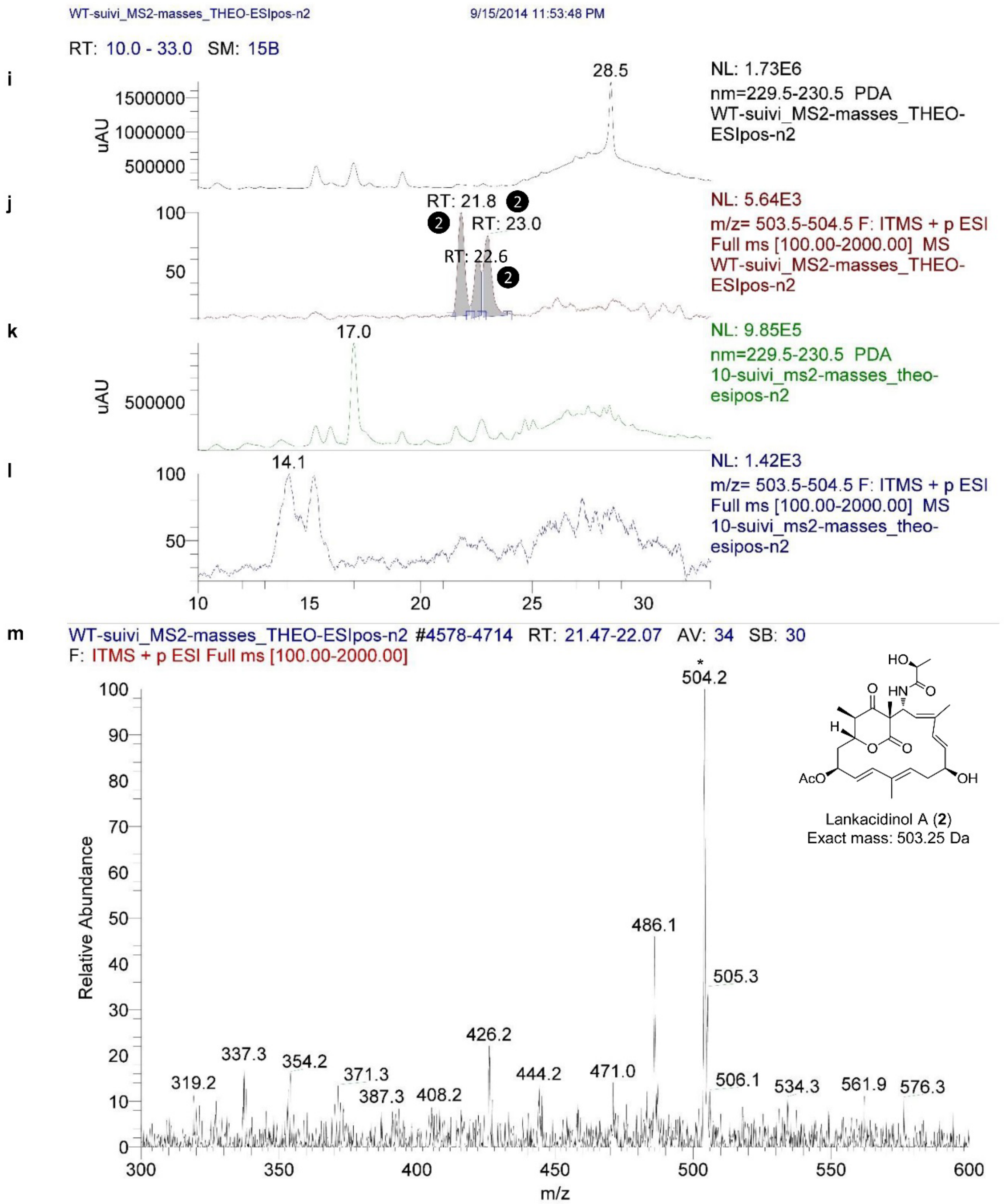

Supplementary Figure 8 │ Analysis by HPLC-MS of extracts of wild type *S. rochei* vs. the Δ*lkcE* inactivation mutant for LC-KA05 (1**) and downstream products, cont.** (**i**)–(**w**) Analysis for metabolites formed downstream of LC-KA05. (**i**)–(**m**) Analysis for lankacidinol A (**2**) (exact mass = 503.25 Da). (**i**) Trace of the absorbance at 230 nm of extracts of the wild type. (**j**) SIM of *m/z* = 504 (ESI positive) of extracts of the wild type. The three peaks corresponding to lankacidinol A are greyed. (**k**) Trace of the absorbance at 230 nm of extracts of the Δ*lkcE* inactivation mutant. (**l**) SIM of *m/z* = 504 (ESI positive) of extracts of the Δ*lkcE* inactivation mutant. As expected for the absence of a functional LkcE, no **2** is detected. (**m**) Mass spectrum of the peak at RT = 21.8 min in (**j**), which is representative of the other two peaks. The parent ion is starred.

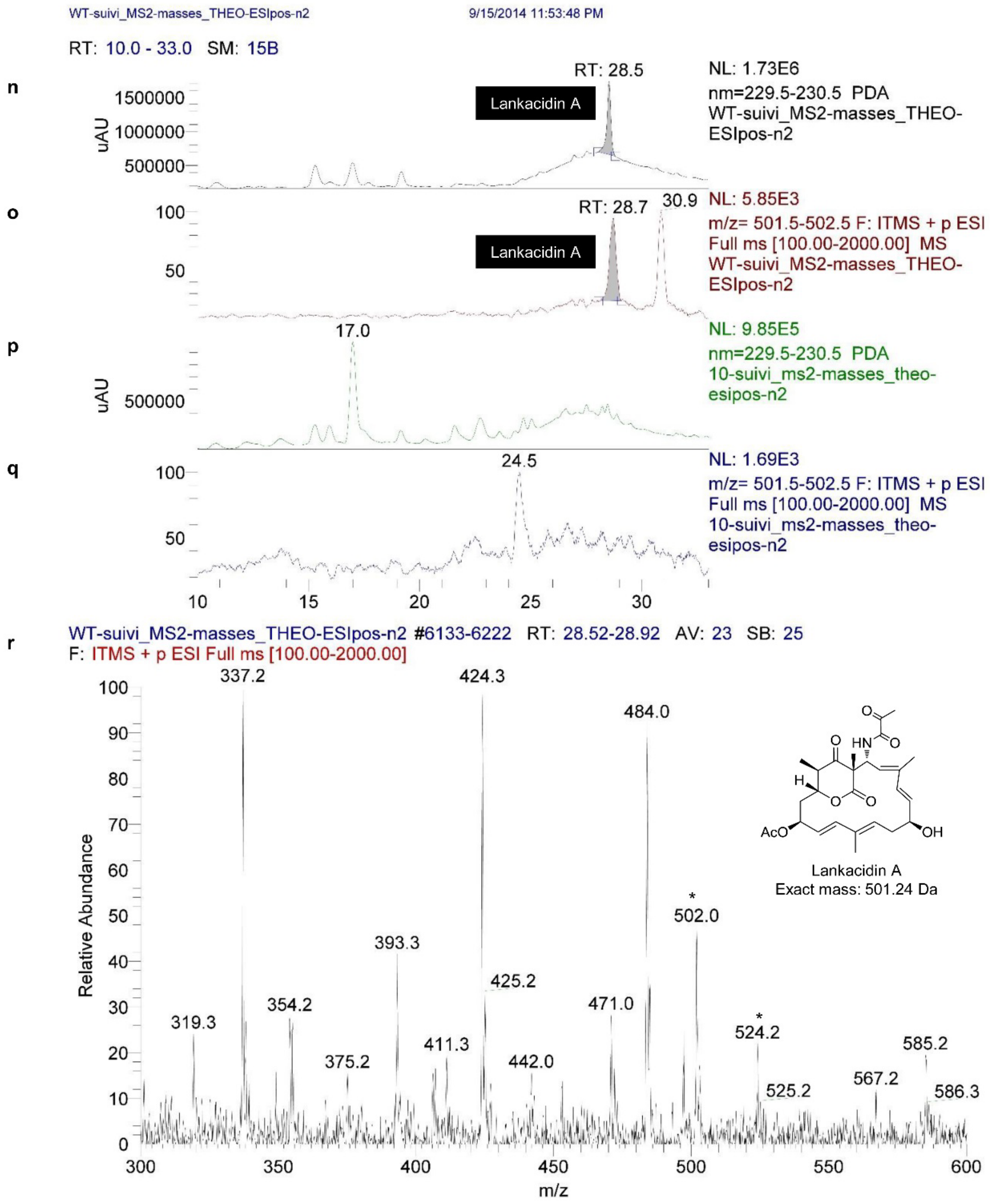

Supplementary Figure 8 │ Analysis by HPLC-MS of extracts of wild type *S. rochei* vs. the Δ*lkcE* inactivation mutant for LC-KA05 (1**) and downstream products, cont.** (**n**) Trace of the absorbance at 230 nm of extracts of the wild type. The peak corresponding to lankacidin A is greyed. (**o**) SIM of m/z = 502 of extracts of the wild type. The peak corresponding to lankacidin A is greyed. (**p**) Trace of the absorbance at 230 nm of extracts of the Δ*lkcE* inactivation mutant. No obvious peak with RT = 28.5 min is present, as expected. (**q**) SIM of m/z = 502 of extracts of the Δ*lkcE* inactivation mutant. No peak with RT = 28.7 min is present, as expected. (**r**) Mass spectrum of the peak at 28.7 min in extracts of the wild type (**o**). Ions consistent with lankacidin A ($[M+H]^+$ = 502.0, $[M+Na]^+$ = 524.1) are starred.

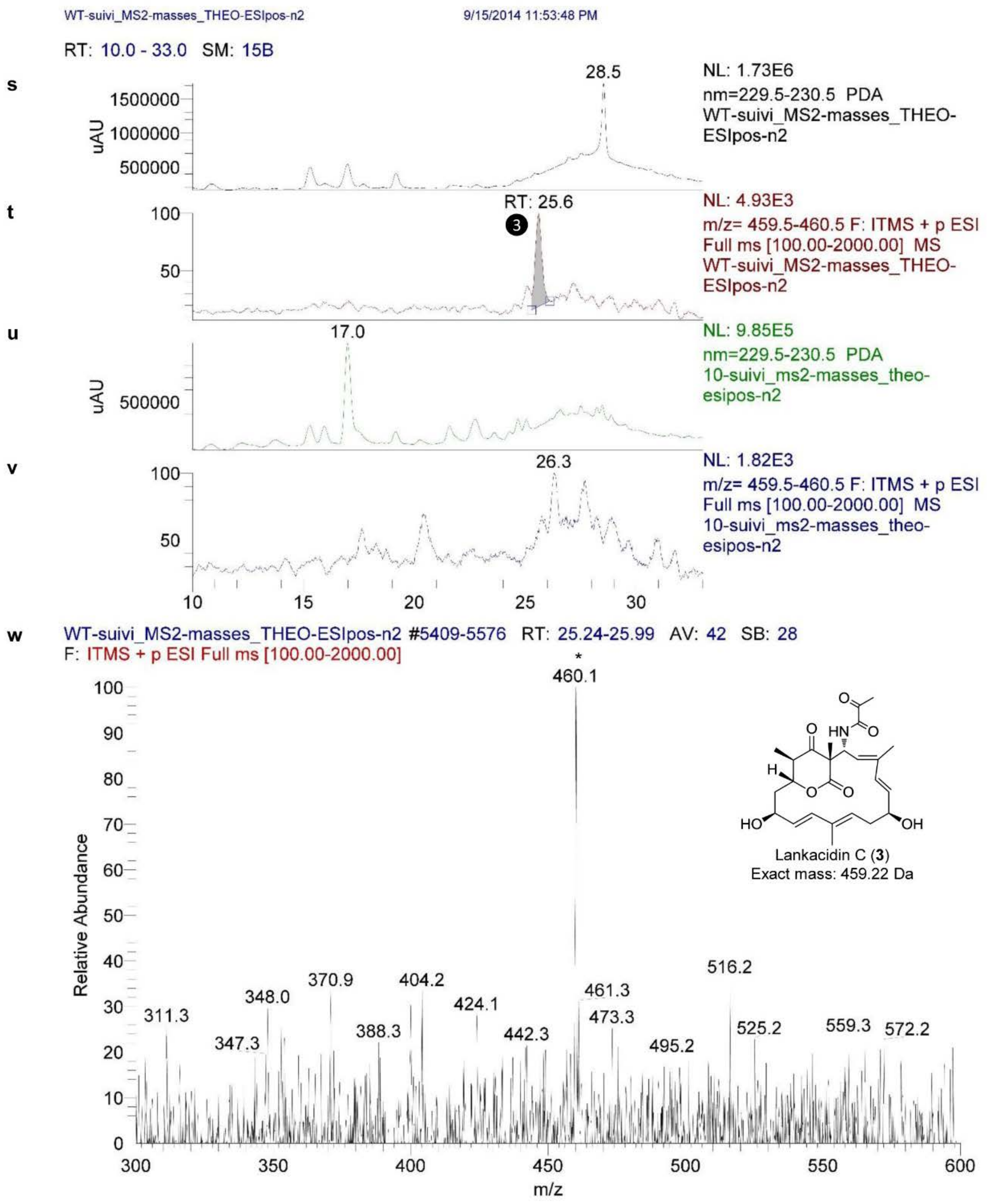

Supplementary Figure 8 │ Analysis by HPLC-MS of extracts of wild type *S. rochei* vs. the Δ*lkcE* inactivation mutant for LC-KA05 (1) and downstream products, cont. (**s**)–(**w**) Analysis for lankacidin C (**3**) (exact mass = 459.22 Da). (**s**) Trace of the absorbance at 230 nm of extracts of the wild type. (**t**) SIM of *m/z* = 460 of extracts of the wild type. The peak corresponding to **3** at RT = 25.6 min is greyed. (**u**) Trace of the absorbance at 230 nm of extracts of the Δ*lkcE* inactivation mutant. (**v**) SIM of *m/z* = 460 of extracts of the Δ*lkcE* inactivation mutant. No peak with RT = 25.6 min is detected, as expected. (**w**) Mass spectrum of the peak at RT = 25.7 min in extracts of the wild type (**t**), showing the expected mass (($[M+H]^+$ = 460.1)) is present (starred).

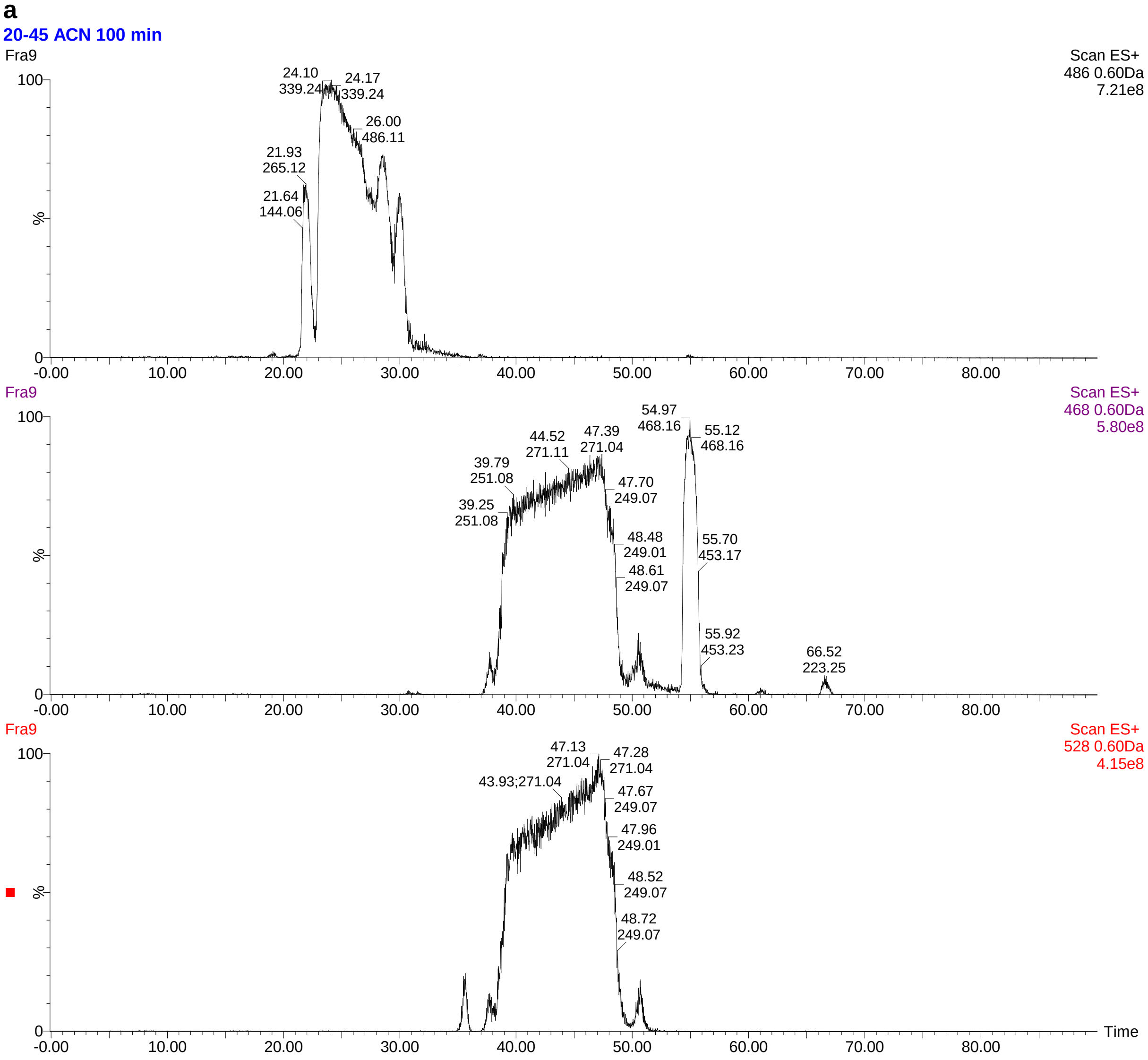
a
20-45 ACN 100 min
Fra9
Scan ES+
486 0.60Da
7.21e8
24.10
339.24
24.17
339.24
26.00
486.11
21.93
265.12
21.64
144.06
%
Fra9
Scan ES+
468 0.60Da
5.80e8
54.97
468.16
55.12
468.16
44.52
271.11
47.39
271.04
39.79
251.08
47.70
249.07
39.25
251.08
48.48
249.01
55.70
453.17
48.61
249.07
55.92
453.23
66.52
223.25
Fra9
Scan ES+
528 0.60Da
4.15e8
47.13
271.04
47.28
271.04
43.93;271.04
47.67
249.07
47.96
249.01
48.52
249.07
48.72
249.07
Time

b

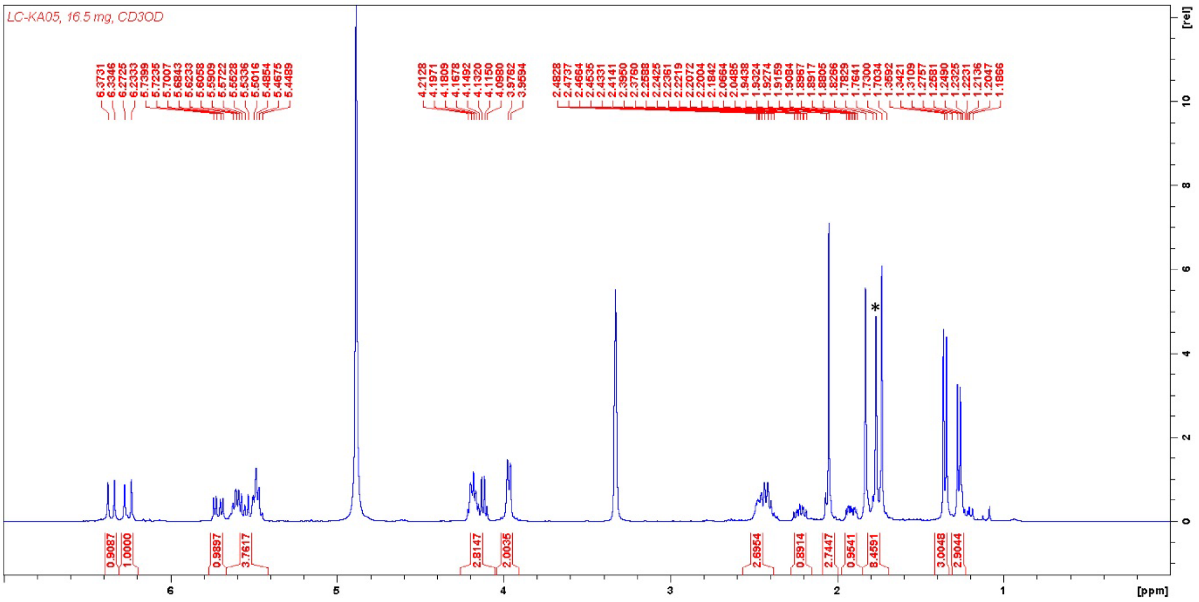

c

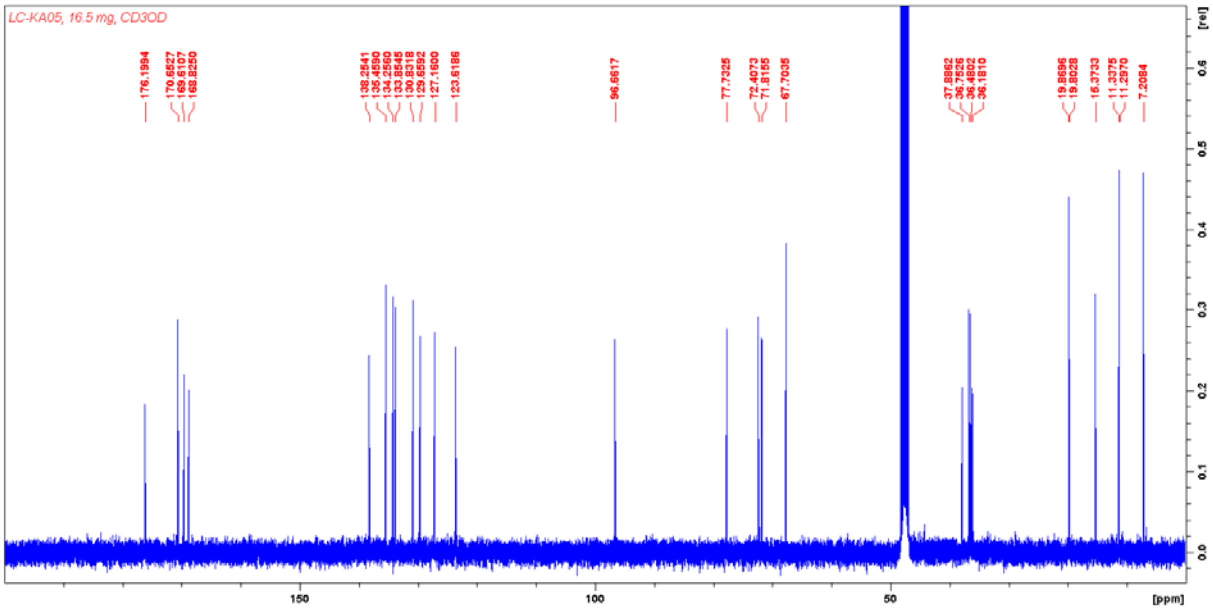

d

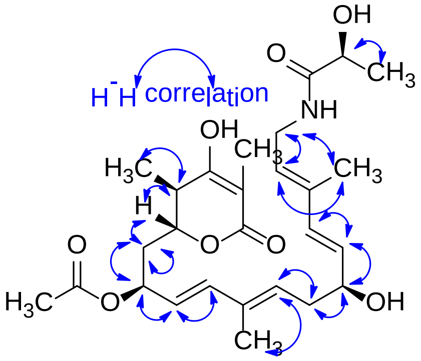
H-H correlation
OH
O
CH3
NH
OH
CH3
H3C
CH3
H
O
O
O
H3C
O
OH
CH3

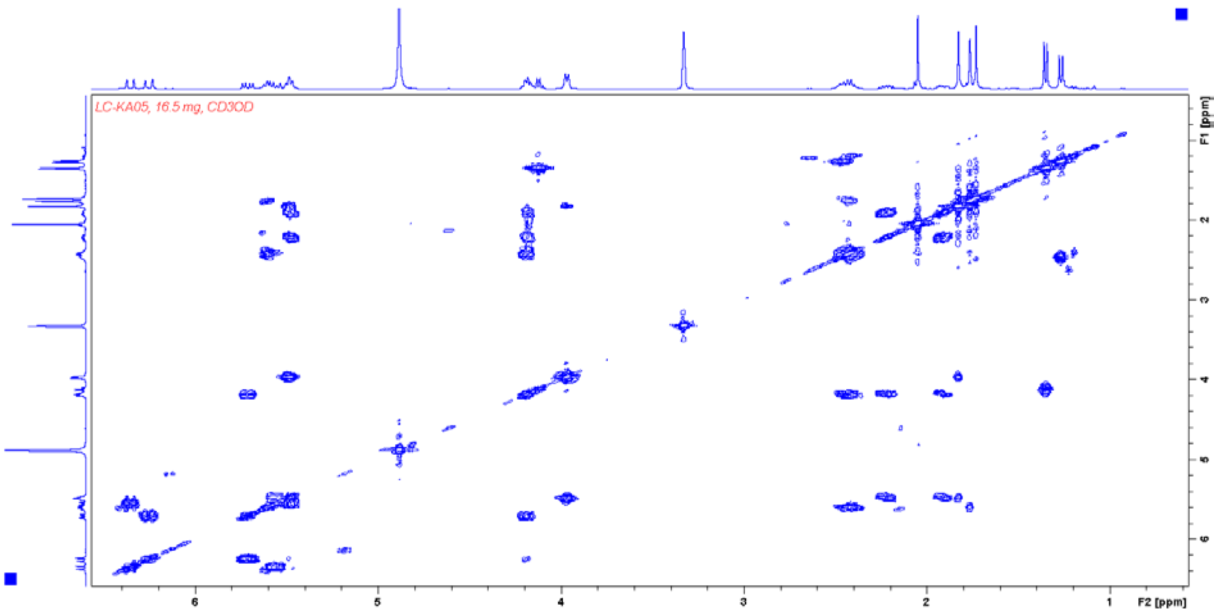
LC-KA05, 16.5 mg, CD3OD
F1 [ppm]
F2 [ppm]

e

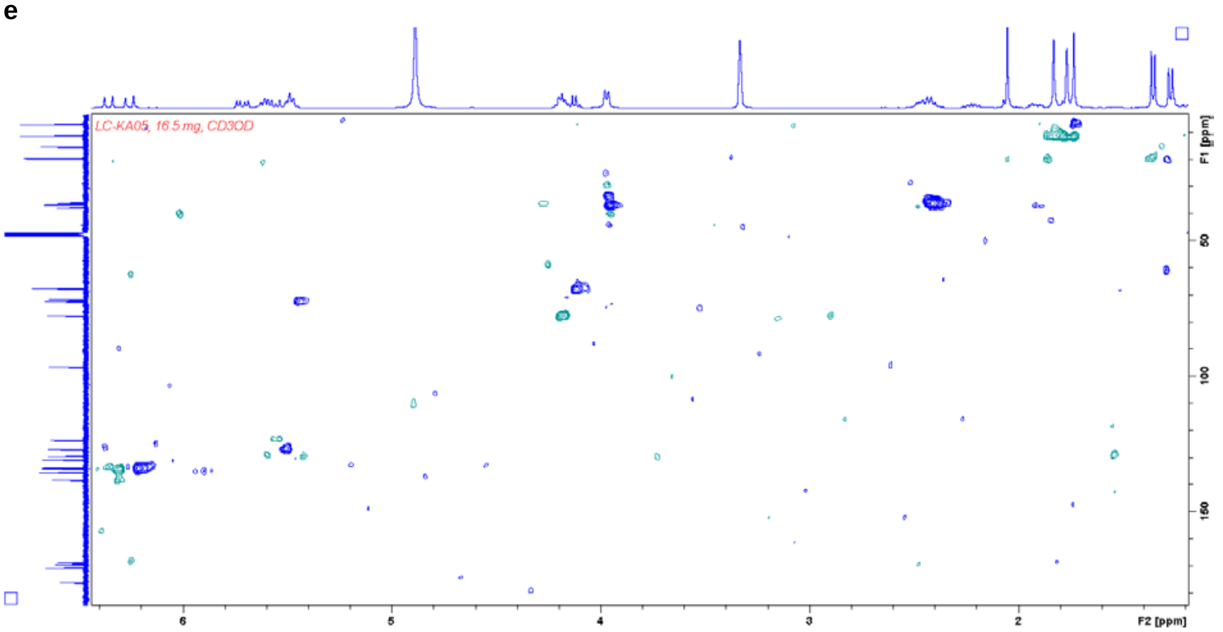
LC-KA05, 16.5 mg, CD3OD
F1 [ppm]
F2 [ppm]

f

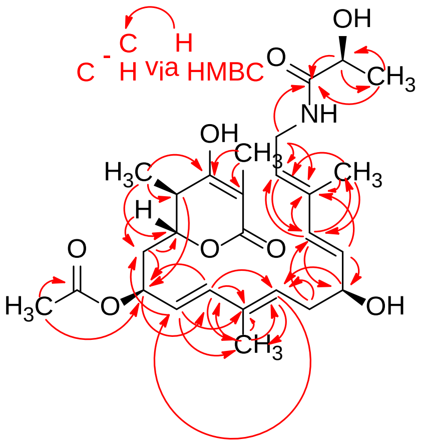
C - H via HMBC
OH
O
CH3
NH
OH
H3C
H
CH3
O
O
H3C
O
OH
CH3

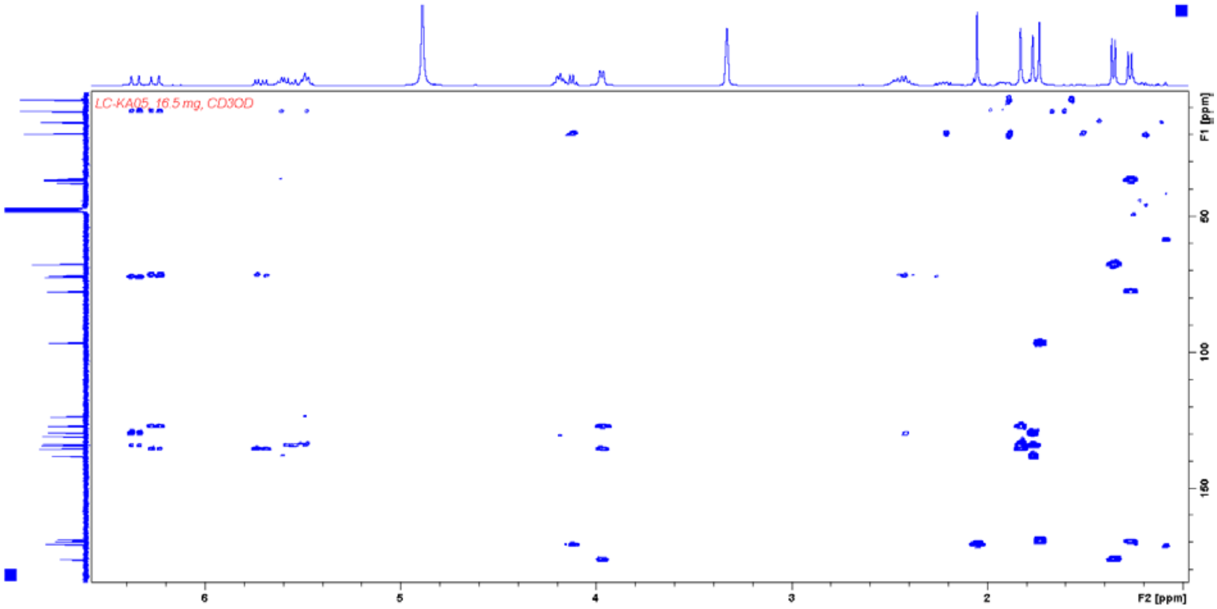
LC-KA05, 16.5 mg, CD3OD
F1 [ppm]
50
100
150
6
5
4
3
2
F2 [ppm]

g

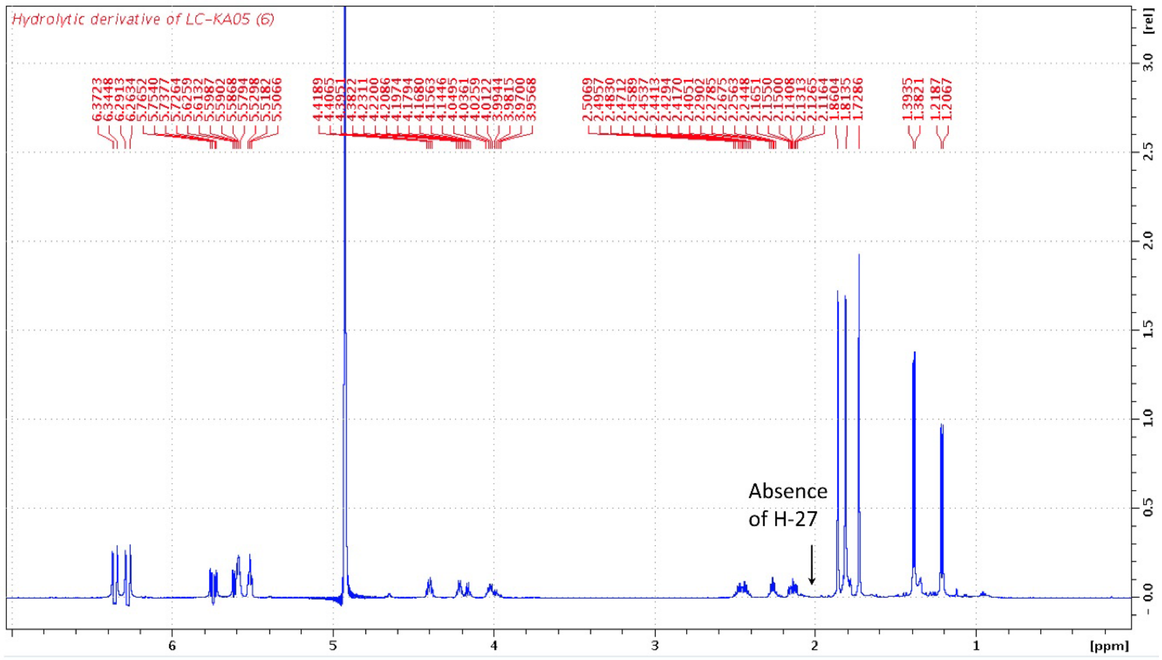

h

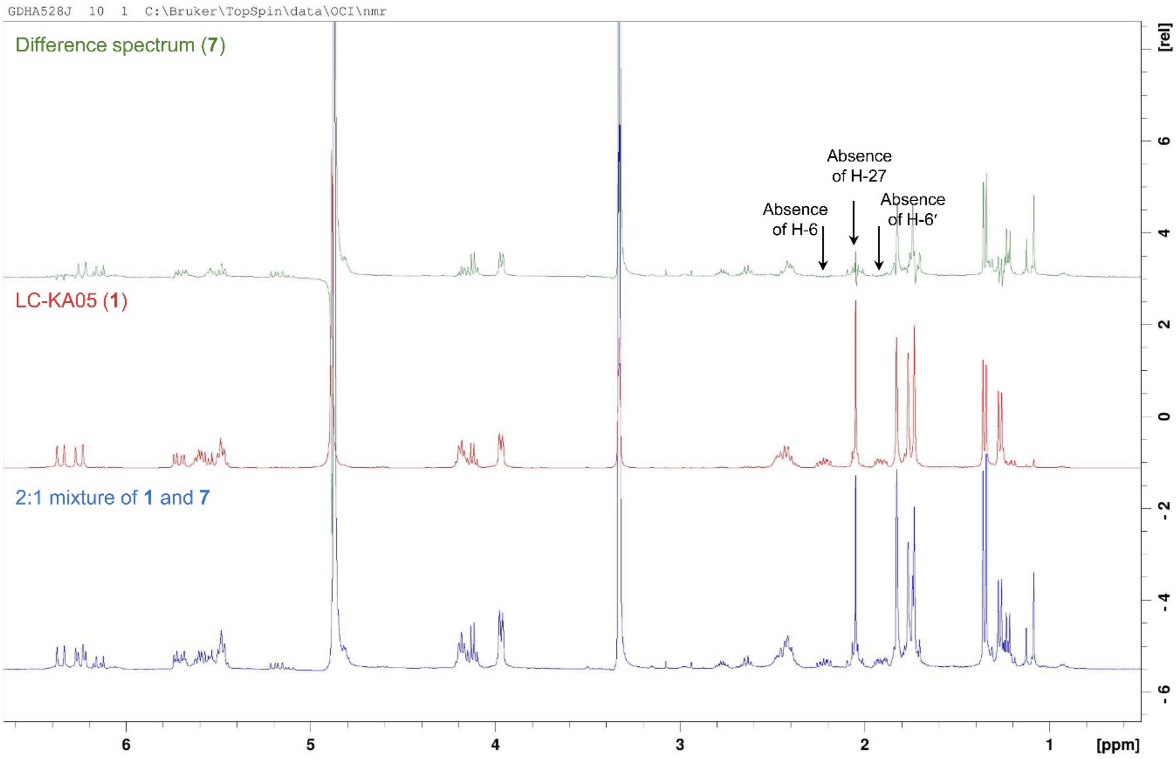

Supplementary Figure 9 │Purification and NMR analysis of LC-KA05 (1**) and its elimination product (**7**).** (**a**) Preparative HPLC-MS of crude extract of *S. rochei* containing LC-KA05. Isolated fractions: RT = 23–27 min (7-OH form **6**; $[M+Na]^+$ *m*/*z* = 486) and 39–48 min (LC-KA05, $[M+Na]^+$ *m*/*z* = 528). The mass of 468 $[M+Na]^+$ corresponds to the elimination product **7** (loss of acetic acid from LC-KA05).This mass is visible as an ionization-induced fragment of LC-KA05, and additionally as a discrete compound eluting at 55 min. (**b**) ^{1}H NMR spectrum of LC-KA05 (in CD_3OD). The * indicates the signal arising from C-19 methyl group, which being a singlet, is diagnostic for the enol form of the compound (the alternative keto form would have given rise to a doublet). In addition, the proton H-2 is

absent from the spectrum. (**c**) ^{13}C NMR spectrum of LC-KA05 (in CD_3OD), which is also consistent with the absence of a ketone group at C-3 and the presence of the C-2–C-3 double bond. (**d**) H-H COSY NMR spectrum of LC-KA05 (in CD_3OD). The structure indicates the observed correlations. (**e**) [^{1}H, ^{15}N]-HSQC NMR spectrum of LC-KA05 (in CD_3OD). (**f**) HMBC spectrum of LC-KA05 (in CD_3OD). The structure indicates the observed correlations. (**g**) ^{1}H NMR spectrum of the 7-OH form **6** (in CD_3OD). The spectrum is overall closely similar to that of LC-KA05 in (**b**), but notably shows the absence of a singlet at ca. 2 ppm corresponding to the three C-27 protons (H-27). (**h**) Comparative NMR analysis of **1** and **7**. In blue, the ^{1}H NMR spectrum of a 2:1 mixture of **1** and **7**. In red, the ^{1}H NMR spectrum of essentially pure **1** (a small amount of this material was obtained, but later decomposed during an attempt at complete purification). The difference spectrum (in green), shows that **7** lacks the signals for the protons H-6 and H-6′ as expected, as well as the three C-27 protons (H-27). Additional new signals appear in the difference spectrum, but these could not be confidently assigned.

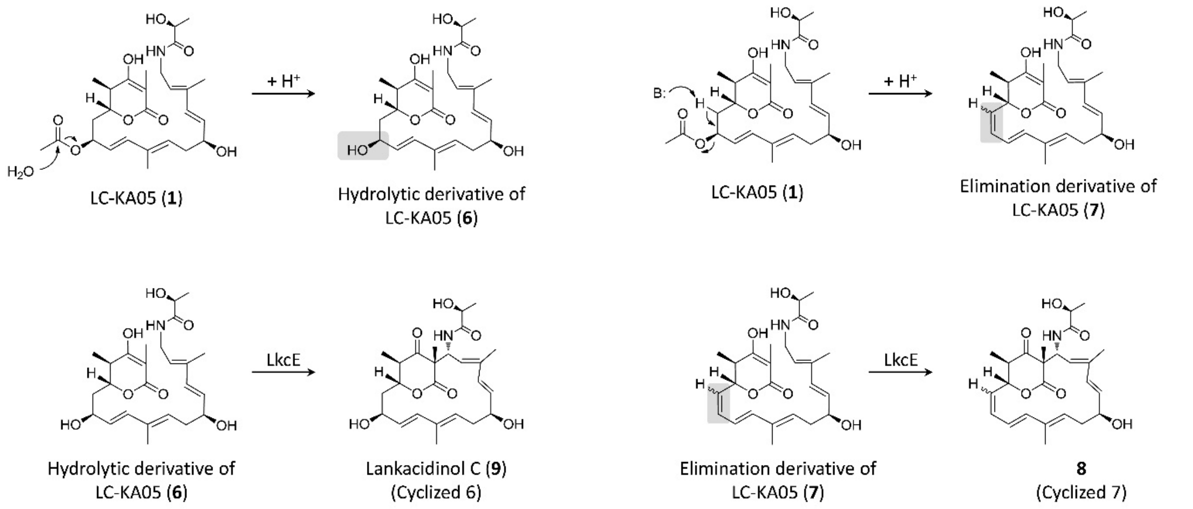

Supplementary Figure 10 │ Analysis by HPLC-MS of extracts of wild type *S. rochei* and the Δ*lkcE* inactivation mutant for degradation products of LC-KA05, 6 **and** 7**, and their cyclized equivalents** 8 **and** 9. Scheme indicating how compounds **6**–**9** are formed.

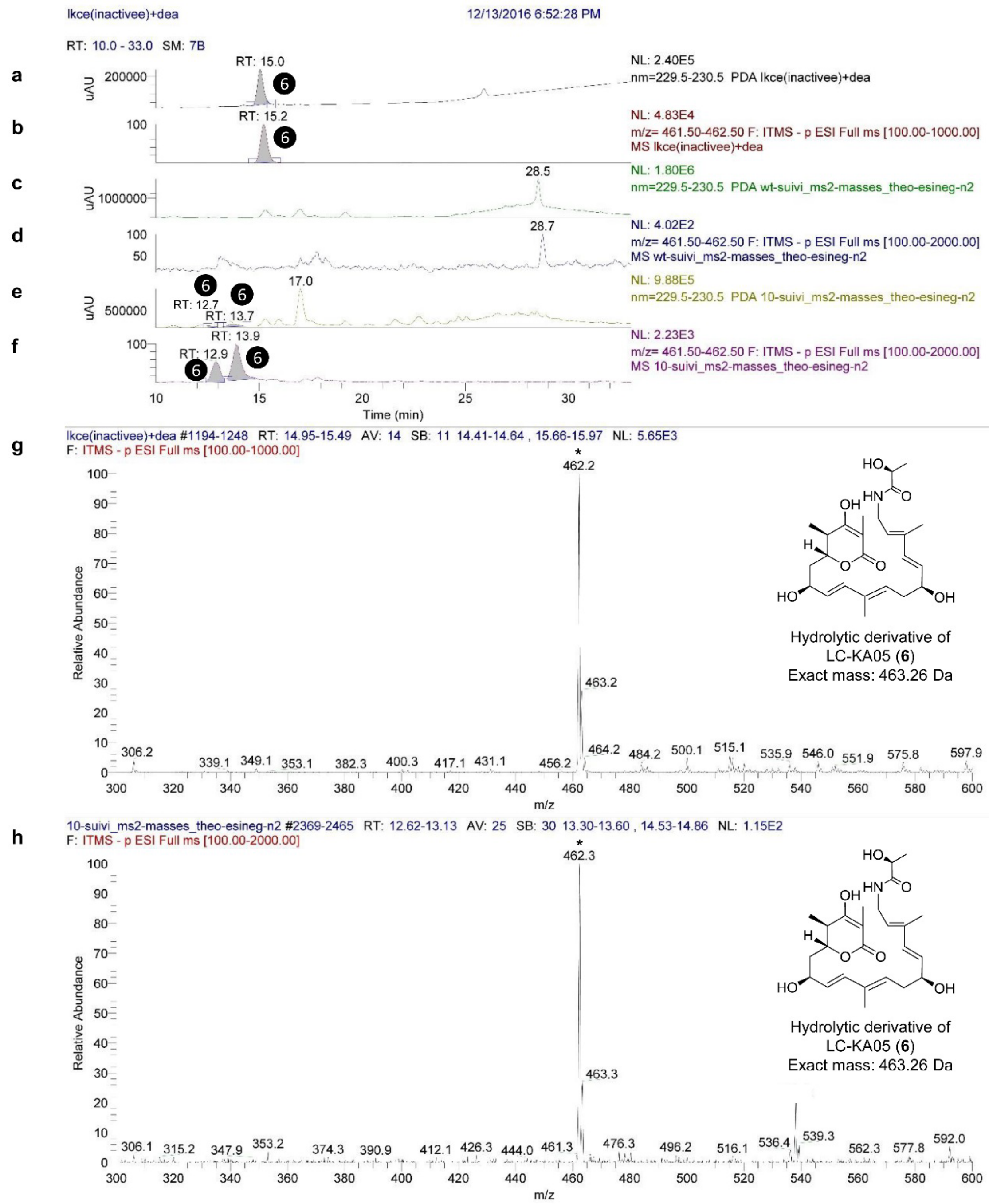

Supplementary Figure 10 │ Analysis by HPLC-MS of extracts of wild type *S. rochei* and the Δ*lkcE* inactivation mutant for degradation products of LC-KA05, 6 **and** 7**, and their cyclized equivalents** 8 **and** 9, **cont.** *m/z* values are reported to one significant figure. (**a**) Control for **6**, the deacetylated (7-OH) derivative of LC-KA05 (exact mass = 463.26 Da). Analysis of extracts of an in vitro assay containing inactivated LkcE and purified **6**. Trace of the absorbance at 230 nm, showing a single peak at RT = 15.0 min corresponding to **6** (greyed). (**b**) Analysis at *m/z* = 462 (ESI negative) of the same in vitro assay yields a single peak at RT = 15.2 min (greyed). (**c**) Trace of the absorbance at 230 nm of extracts of wild type *S. rochei*, showing the absence of a significant peak at RT = 15.0 min. (**d**) Analysis of wild type *S. rochei* at *m/z* = 462 (ESI negative) also did not reveal any peak corresponding to **6**. (**e**) Trace of the absorbance at 230 nm of extracts of the Δ*lkcE* inactivation mutant. Two candidate peaks corresponding to **6** are observed (greyed). (**f**) Analysis of the Δ*lkcE* inactivation mutant at *m/z* = 462 (ESI negative) reveals two candidate peaks corresponding to **6** with the same RT as in (**e**) (RT = 12.9 and 13.9). The identity of the peaks as **6** are supported by their mass spectra (see (**h**) for a representative spectrum). (**g**) Mass spectrum of the peak at 15.4 min in (**b**). The parent ion is starred. (**h**) Mass spectrum of the peak at RT = 13.9 min in (**f**) which is representative of that at RT = 12.9 min. The parent ion is starred.

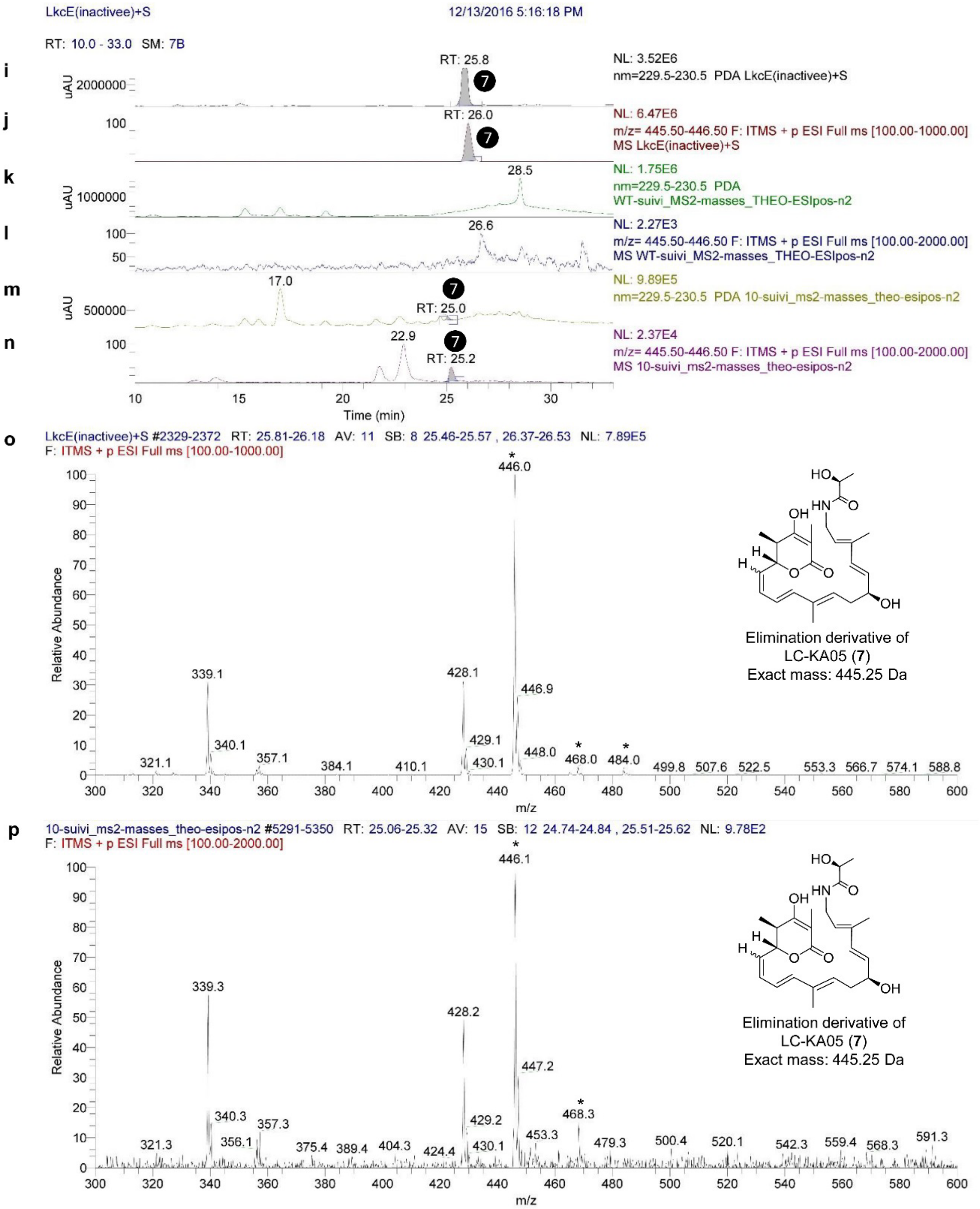

Supplementary Figure 10 │ Analysis by HPLC-MS of extracts of wild type *S. rochei* and the Δ*lkcE* inactivation mutant for degradation products of LC-KA05, 6 **and** 7**, and their cyclized equivalents** 8 **and** 9**, cont.** (**i**) Control for **7**, the acetate eliminated derivative of LC-KA05 (exact mass = 445.25 Da). Trace of the absorbance at 230 nm of an in vitro assay containing inactivated LkcE and the purified, 2:1 mixture of LC-KA05 **1**/eliminated derivative **7**, showing a single peak at RT = 25.8 min corresponding to **7** (greyed). (**j**) Analysis of extracts of the same in vitro assay containing the **1**/**7** mixture. Analysis at *m/z* = 446 (ESI positive) yielded a single peak at RT = 26.0 min. (**k**) Trace of the absorbance at 230 nm of extracts of wild type *S. rochei*. No distinct peak corresponding to **7** is observed. (**l**) Analysis at *m/z* = 446 (ESI positive) of extracts of wild type *S. rochei* does not yield a clear peak corresponding to **7**. (**m**) Trace of the absorbance at 230 nm of extracts of the Δ*lkcE* inactivation mutant, reveals a peak corresponding to **7** at RT = 25.0 min. (**n**) Analysis of the Δ*lkcE* inactivation mutant at *m/z* = 446 (ESI positive) yields the same peak (RT = 25.2 min). (**o**) Mass spectrum of the peak at RT = 26.0 min in (**j**) shows ions consistent with **7** ($[M+H]^+$ = 446.0, $[M+Na]^+$ =

468.0, [M+K]$^+$ = 484.0, starred). (**p**) Mass spectrum of the peak at RT = 25.2 min in (**n**), shows ions consistent with **7** ([M+H]$^+$ = 446.1, [M+Na]$^+$ = 468.3).

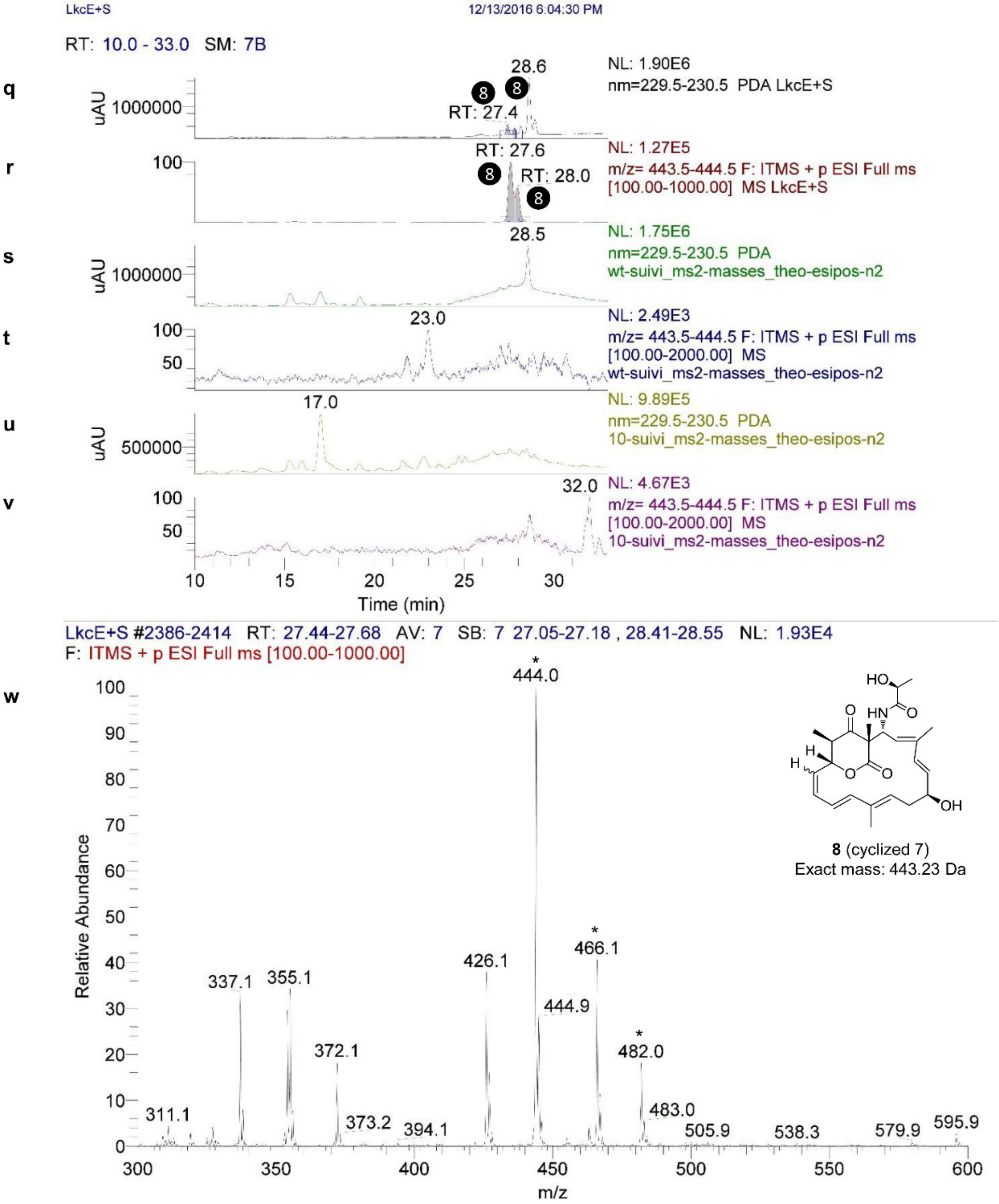

Supplementary Figure 10 │ Analysis by HPLC-MS of extracts of wild type *S. rochei* and the Δ*lkcE* inactivation mutant for degradation products of LC-KA05, 6 **and** 7**, and their cyclized equivalents** 8 **and** 9**, cont.** (**q**) Control for **8** the cyclized form of the elimination derivative 7 (exact mass = 443.23 Da). Analysis of an in vitro assay containing active LkcE and a 2:1 mixture of LC-KA05 (**1**) and the eliminated derivative **7**. Trace of the absorbance at 230 nm yields two candidate peaks for the cyclized product **8** (RT = 27.4 and 28.6 min). (**r**) Analysis of the same in vitro assay at *m/z* = 444 (ESI positive) yields two peaks at essentially the same RT (RT = 27.6 and 28.0 min). (**s**) Trace of the absorbance at 230 nm of extracts of wild type *S. rochei*. No distinct peak corresponding to **8** is observed. (**t**) Analysis at *m/z* = 444 (ESI positive) of extracts of wild type *S. rochei* also does not yield a clear peak corresponding to **8**. (**u**) Trace of the absorbance at 230 nm of extracts of the *lkcE* inactivation mutant. No distinct peak corresponding to **8** is observed. (**v**) Analysis at *m/z* = 444 (ESI positive) of extracts of the *lkcE* inactivation mutant also does not yield a clear peak corresponding to **8**. (**w**) Mass spectrum of the peak at RT = 27.6 in (**r**), showing peaks consistent with **8** ([M+H]$^+$ = 444.0, [M+Na]$^+$ = 466.1, [M+K]$^+$ = 482.0, starred).

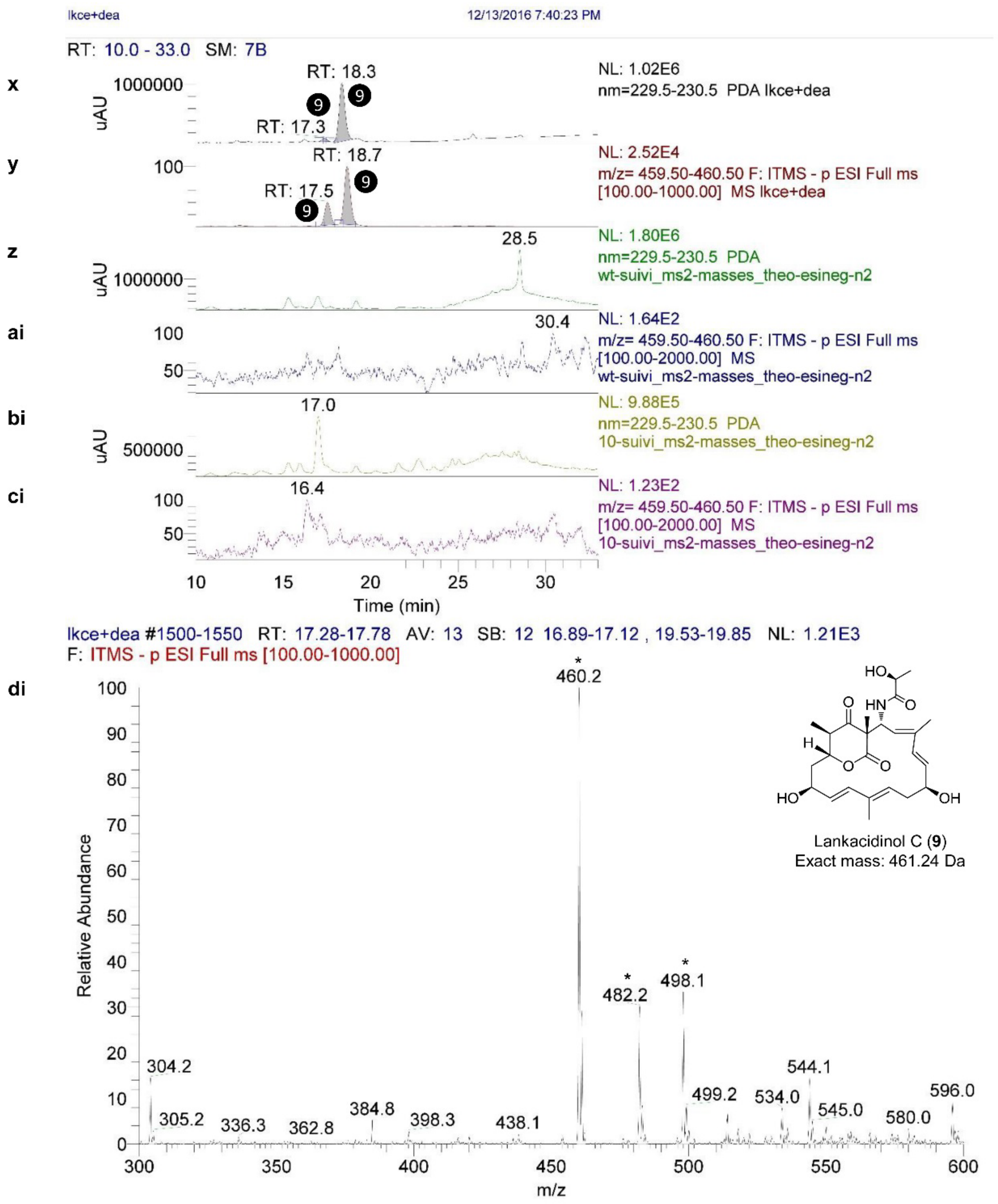

Supplementary Figure 10 │ Analysis by HPLC-MS of extracts of wild type *S. rochei* and the Δ*lkcE* inactivation mutant for degradation products of LC-KA05, 6 **and** 7**, and their cyclized equivalents** 8 **and** 9, **cont.** (**x**) Control for lankacidinol C (**9**), the cyclized form of 6 (exact mass = 461.24 Da). Analysis of extracts of an in vitro assay containing active LkcE and purified 6. Trace of the absorbance at 230 nm, showing two peaks at RT = 17.3 and 18.3 min corresponding to **9** (greyed). (**y**) Analysis at *m/z* = 460 (ESI negative) of the same in vitro assays also yields two peaks (RT = 17.5 and 18.7 min). (**z**) Trace of the absorbance at 230 nm of extracts of wild type *S. rochei*, showing the absence of peaks corresponding to **9**. (**ai**) Analysis of extracts of wild type *S. rochei* at *m/z* = 460 (ESI negative) also did not reveal any peak corresponding to **9**. (**bi**) Trace of the absorbance at 230 nm of extracts of Δ*lkcE* inactivation mutant, showing the absence of peaks corresponding to **9**, as expected. (**ci**) Analysis of extracts of Δ*lkcE* inactivation mutant at *m/z* = 460 (ESI negative) also did not reveal any peak corresponding to **9**. (**di**) Mass spectrum of the peak at RT = 17.5 in (**y**), showing peaks consistent with **9** ($[M–H]^–$ = 460.2, $[M–2H+Na]^–$ = 482.2, $[M–2H+K]^–$ = 498.1, starred).

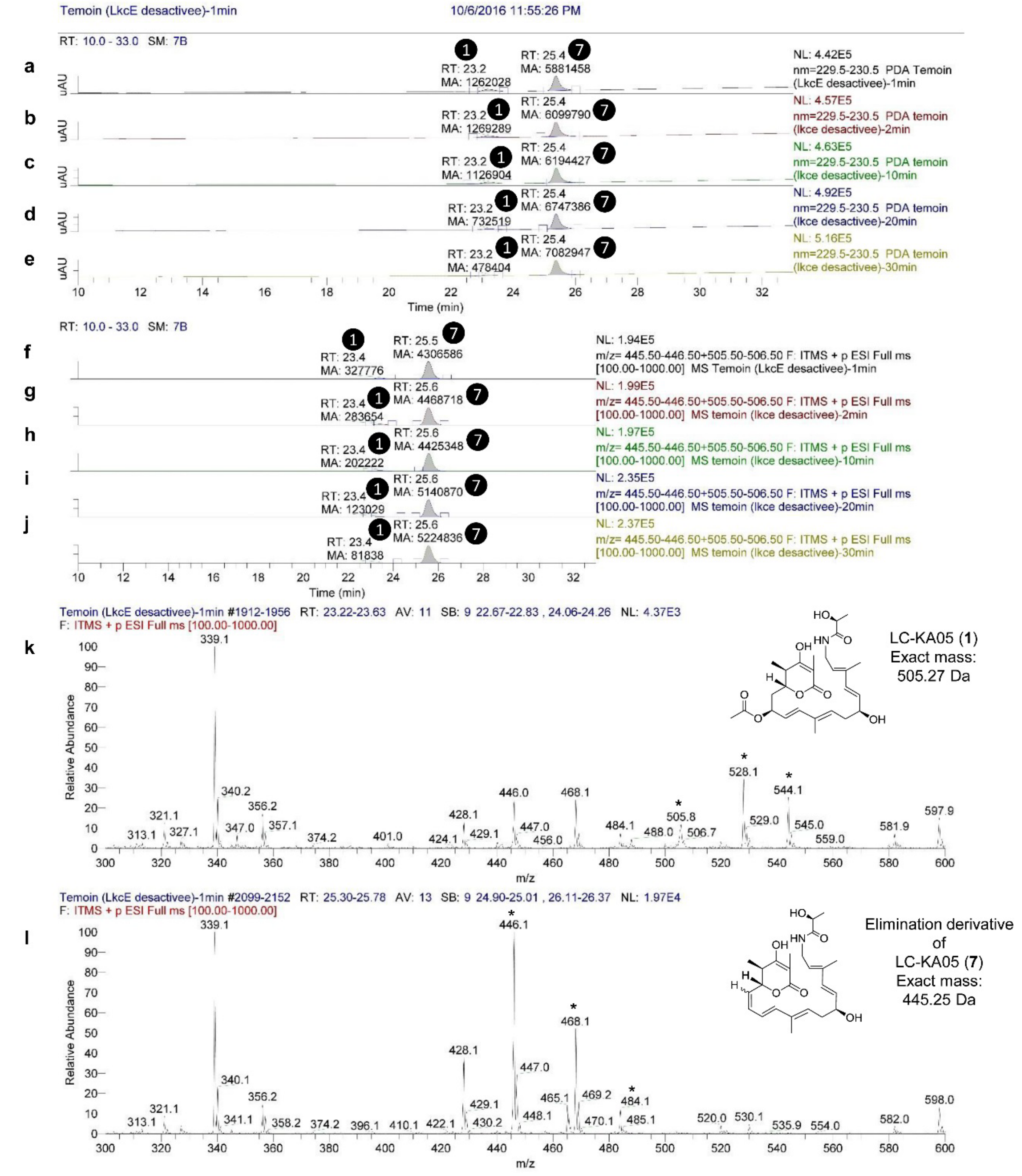

Supplementary Figure 11 │ Analysis by HPLC-MS of the stability of LC-KA05 under standard LkcE in vitro assay conditions. Incubations were carried out in the presence of inactivated LkcE and the 2:1 mixture of LC-KA05 (**1**)/eliminated derivative **7**, and the substrates extracted and analyzed at various time points. (**a**)–(**e**) Trace of the absorbance at 230 nm showing peaks at RT = 23.2 and 25.4 min, corresponding to **1** and **7**, respectively. Integration of the peak areas shows that even in the absence of reaction, **1** decreased over time (1–30 min), while **7** increased correspondingly. (**f**)–(**j**) Analysis of the same time points at *m/z* = 446 (ESI positive) (for **7**) and *m/z* = 506 (for **1**). This analysis produced peaks with essentially the same retention times as in (**a**)–(**e**), and which showed the same behavior in terms of change in area with time. (**k**) Mass spectrum of the peak at RT = 23.4 min in (**f**) corresponding to **1**. Although the retention time differs from that in **Supplementary Fig. 8** (**d**), the mass spectrum contains the same major peaks (including ($[M+H]^+$ = 505.8, $[M+Na]^+$ = 528.1, $[M+K]^+$ = 544.1, starred). The difference in RT may be explained by the fact that **1** was analyzed within an extract in **Supplementary Fig. 8** (**d**), while the analysis here was carried out on purified compound following in vitro assay (and see **Table S4**). (**l**) Mass spectrum of the peak at RT = 25.5 min in (**f**) corresponding to **7** (for comparison with **Supplementary Fig. 10** (**o**)).

Control reaction with **1/7** mixture

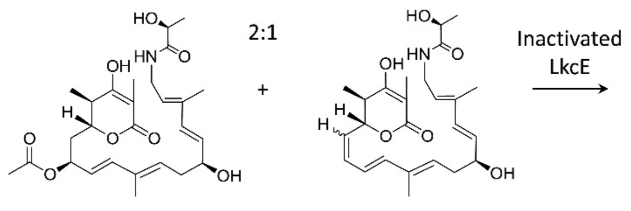

LC-KA05 (**1**)

Elimination derivative of LC-KA05 (**7**)

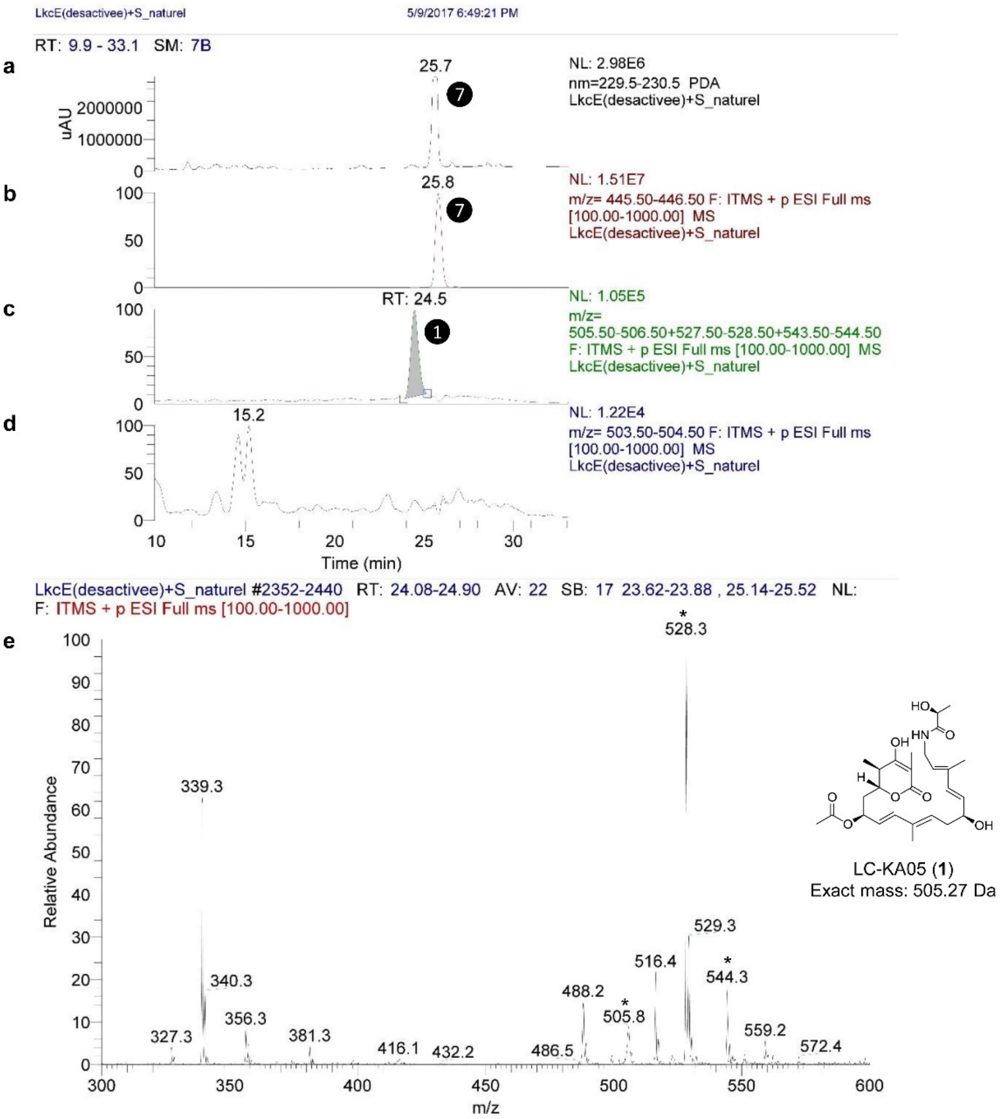

Supplementary Figure 12 │ Demonstration by HPLC-MS that LkcE is active in vitro. A reaction scheme is provided for each experiment. (**a**) Trace of the absorbance at 230 nm of a control reaction (24 h incubation) with inactivated LkcE and the 2:1 native substrate LC-KA05 (**1**) (exact mass = 505.27 Da)/eliminated derivative **7** (exact mass = 445.25 Da) mixture, showing a peak (RT = 25.7 min) corresponding to **7**. (**b**) Analysis of the control reaction at *m/z* = 446 (ESI positive), gives a peak for **7** at essentially the same retention time as in (**a**) (RT = 25.8 min). (**c**) Analysis of the control reaction at *m/z* = 506 (ESI positive) for **1**, yields a peak at RT = 24.5 min. (**d**) Analysis of the control reaction for *m/z* = 504 corresponding to the expected mass of lankacidinol A (**2**) (exact mass = 503.25 Da), does not yield a peak with the appropriate retention time (20–27 min, **Supplementary Table 4**). (**e**) Mass spectrum of the peak at 24.5 min in (**c**), showing peaks characteristic of **1** ($[M+H]^+$ = 505.8, $[M+Na]^+$ = 528.3, $[M+K]^+$ = 544.3, starred) (and see **Supplementary Table 4**).

Assay of active LkcE with **1**/**7** mixture

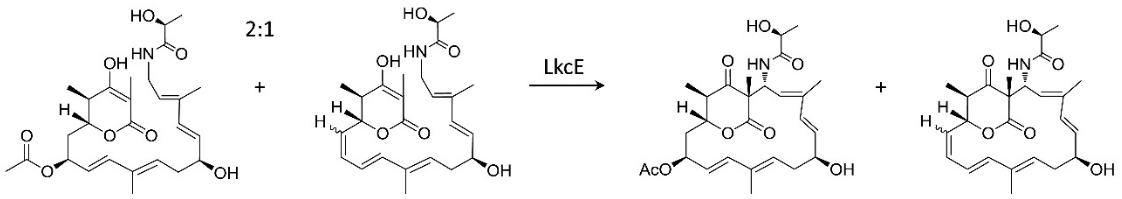

LC-KA05 (**1**) | Elimination derivative of LC-KA05 (**7**) | Lankacidinol A (**2**) | **8** (Cyclized 7)

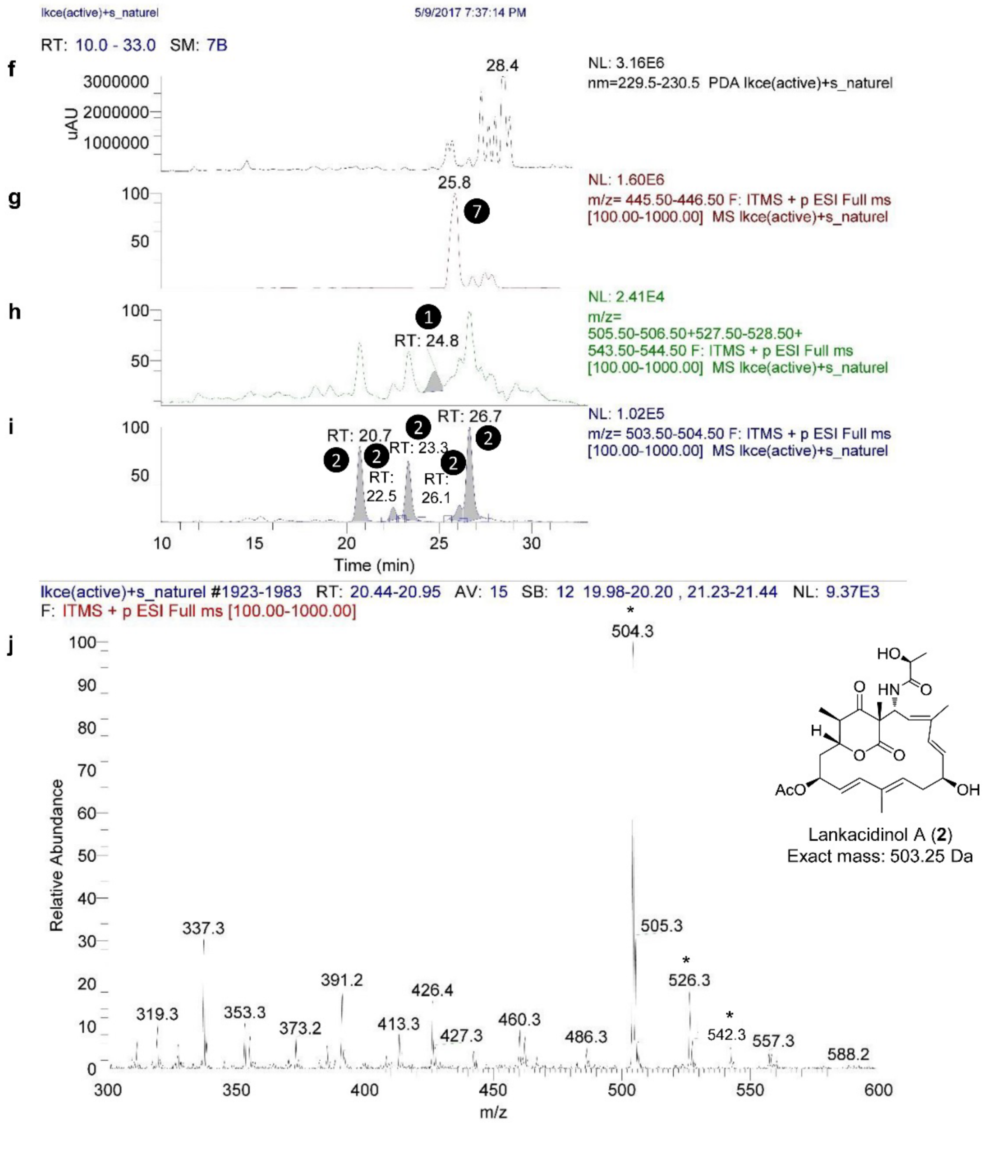

Supplementary Figure 12 │ Demonstration by HPLC-MS that LkcE is active in vitro, cont. (**f**) Trace of the absorbance at 230 nm of a reaction with active LkcE and the 2:1 **1**/ **7** mixture, after 24 h incubation. (**g**) Analysis of the LkcE reaction at *m/z* = 446 (ESI positive) yields a peak corresponding to unreacted **7**. (**h**) Analysis of the LkcE reaction at *m/z* = 506 (ESI positive) for **1**, yields a peak at RT = 24.8 min corresponding to unreacted **1**. (**i**) Analysis of the LkcE reaction at *m/z* = 504 for **2**, yields 5 peaks (RT = 20.7, 22.5, 23.3, 26.1 and 26.7 min) which are notably not present in the control reaction (**d**). (**j**) Mass spectrum of the peak in (**i**) at RT = 20.7 min, which is representative of the others. This shows peaks characteristic of **2** ($[M+H]^+$ = 504.3, $[M+Na]^+$ = 526.3, $[M+K]^+$ = 542.3, starred).

Assay of active LkcE with **1**/**7** mixture

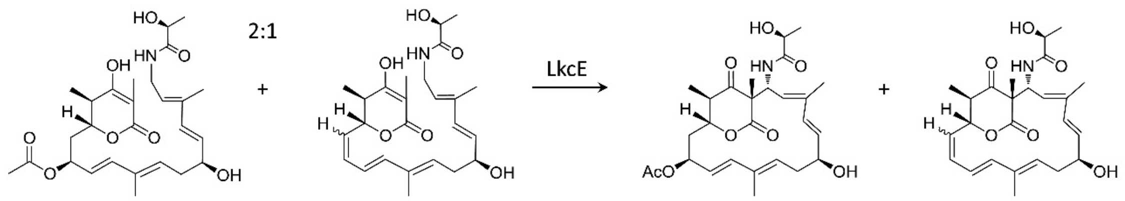

LC-KA05 (**1**) Elimination derivative of LC-KA05 (**7**) Lankacidinol A (**2**) **8** (Cyclized 7)

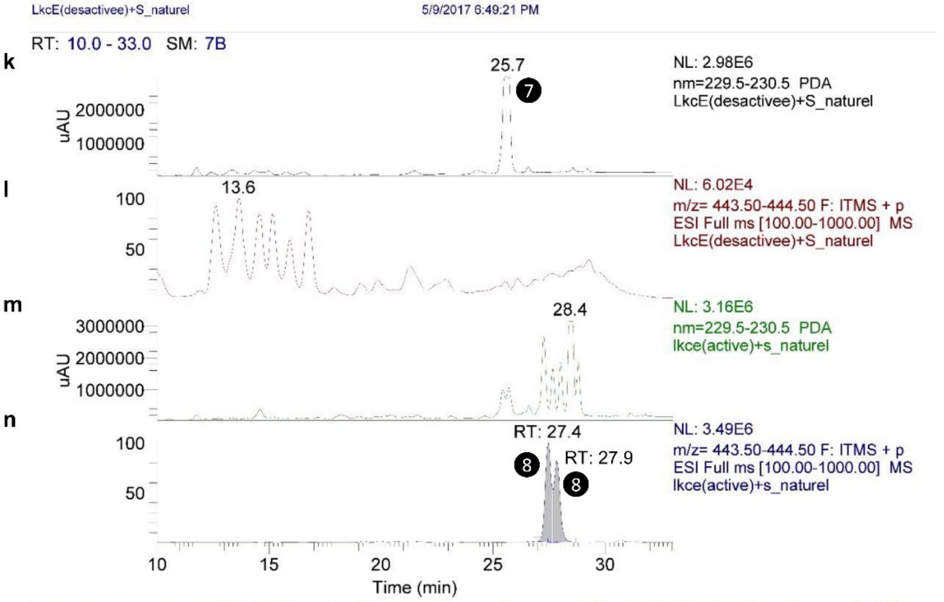

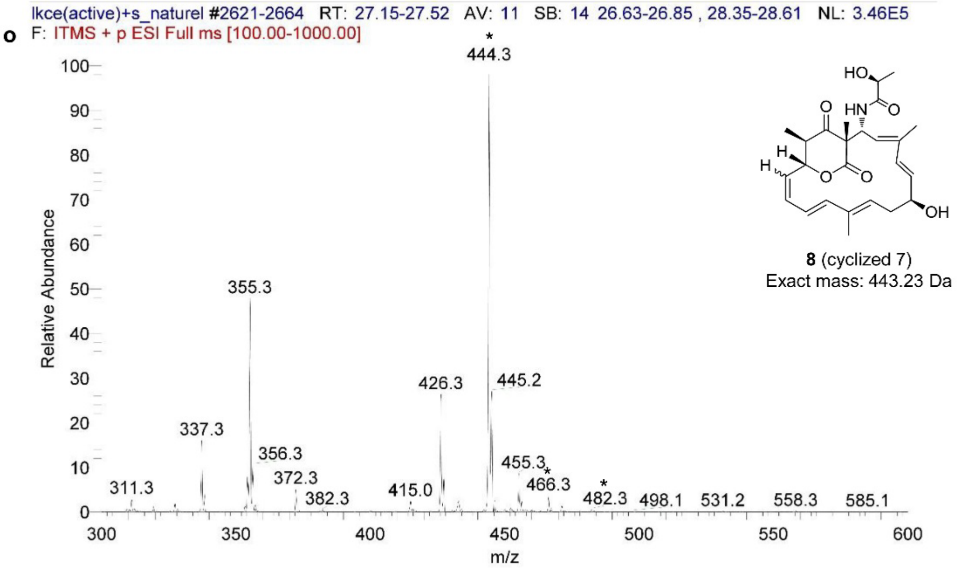

Supplementary Figure 12 │ Demonstration by HPLC-MS that LkcE is active in vitro, cont. (**k**) Trace of the absorbance at 230 nm of a control reaction (24 h incubation) with inactivated LkcE and the 2:1 **1**/**7** mixture, showing a peak (RT = 25.7 min) corresponding to **7**. (**l**) Analysis of the control reaction at *m/z* = 444 (ESI positive) corresponding to **8** (cyclized 7), does not yield a candidate peak. (**m**) Trace of the absorbance at 230 nm of a reaction with active LkcE and the 2:1 **1**/ **7** mixture, after 24 h incubation, yields multiple candidate peaks for **8** in the 27–28 min range, which are not present in (**l**). (**n**) Analysis of the LkcE reaction at *m/z* = 444 for **8**, yields 2 peaks (RT = 27.4 and 27.9 min) which are notably not present in the control reaction (**d**). (**o**) Mass spectrum of the peak at 27.4 min which is

representative of the two, showing ions consistent with **8** ([M+H]$^+$ = 444.3, [M+Na]$^+$ = 466.3, [M+K]$^+$ = 482.3, starred).

Control reaction (panels **p**–**r**)

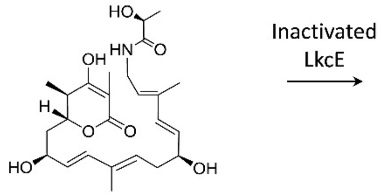

Hydrolytic derivative of
LC-KA05 (**6**)

Assay of active LkcE with **6** (panels **s**–**v**)

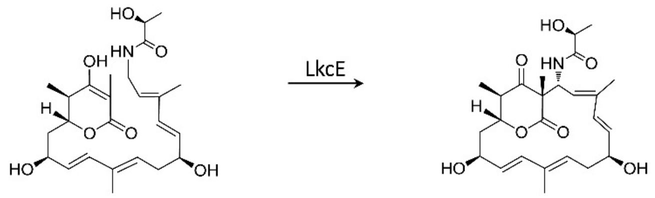

Hydrolytic derivative of
LC-KA05 (**6**)

Lankacidinol C (**9**)
(Cyclized 6)

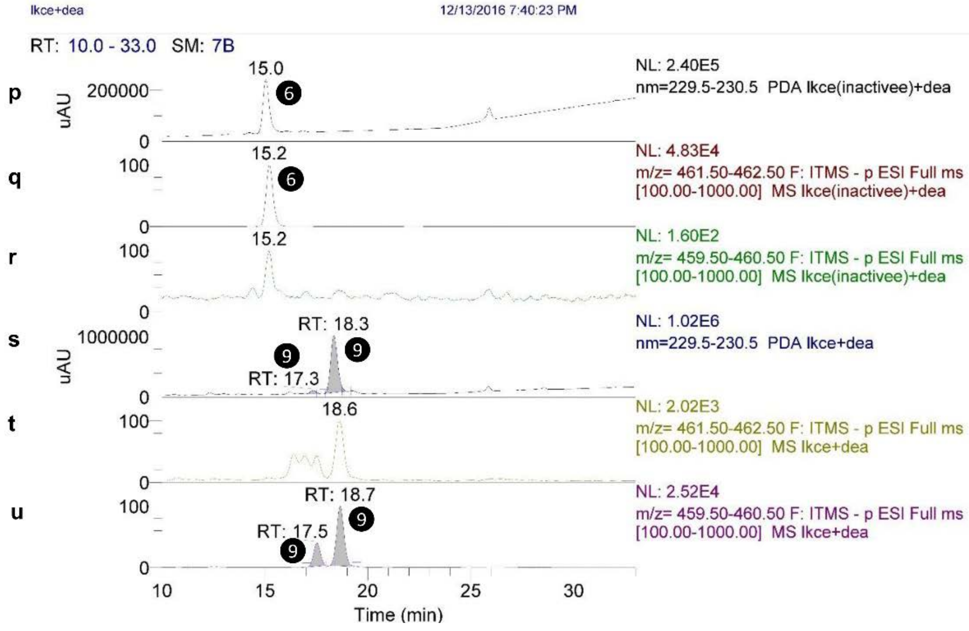

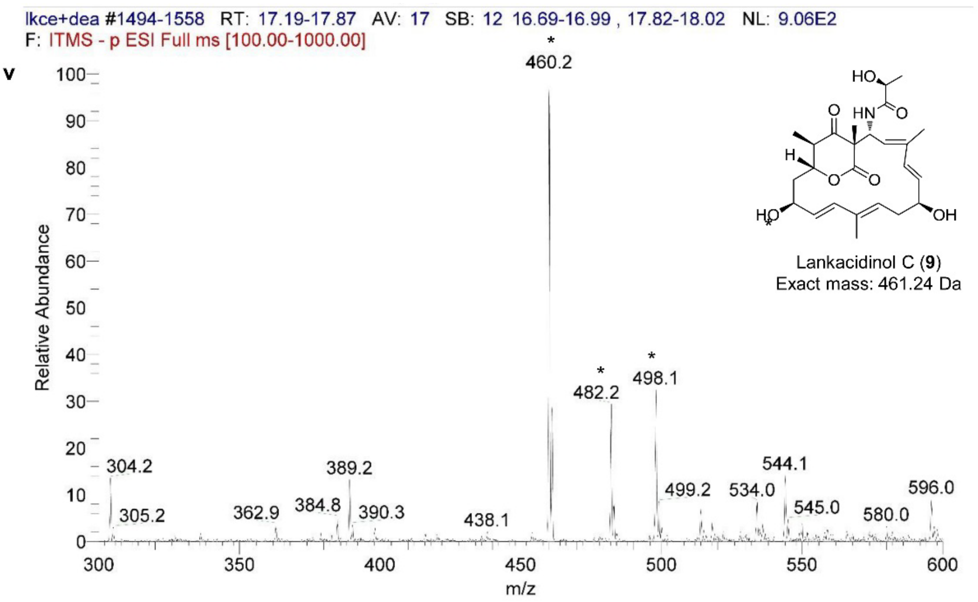

Supplementary Figure 12 │ Demonstration by HPLC-MS that LkcE is active in vitro, cont. (**p**) Trace of the absorbance at 230 nm of a control reaction (24 h incubation) with inactivated LkcE and purified **6**, the deacetylated (7-OH) derivative of LC-KA05 (exact mass = 463.26 Da), showing a peak corresponding to unreacted **6** (RT = 15.0 min). (**q**) Analysis of the control reaction at *m/z* = 462 (ESI negative) for unreacted **6**, gives a peak at the same RT. (**r**) Analysis of the control reaction at *m/z* = 460 for lankacidinol C (**9**) (cyclized 6) does not yield a candidate peak. (**s**) Trace of the absorbance at 230 nm of a reaction with active LkcE and purified **6**, yields two peaks not present in the control reaction (**p**) (RT = 17.3 and 18.4 min). (**t**) Analysis of the LkcE reaction at *m/z* = 462 (ESI negative) for unreacted **6** does not yield a peak at RT =15 min, as observed in the control. (**u**) Analysis of the LkcE reaction at *m/z* = 460 for **9**, yields the two peaks not present in the control (RT = 17.5 and 18.7 min). (**v**) Mass spectrum (ESI negative) of the peak at RT = 17.5 in (**u**) yields peaks consistent with **9** ($[M{-}H]^-$ = 460.2, $[M{-}2H{+}Na]^-$ = 482.2, $[M{-}2H{+}K]^-$ = 498.1, starred).

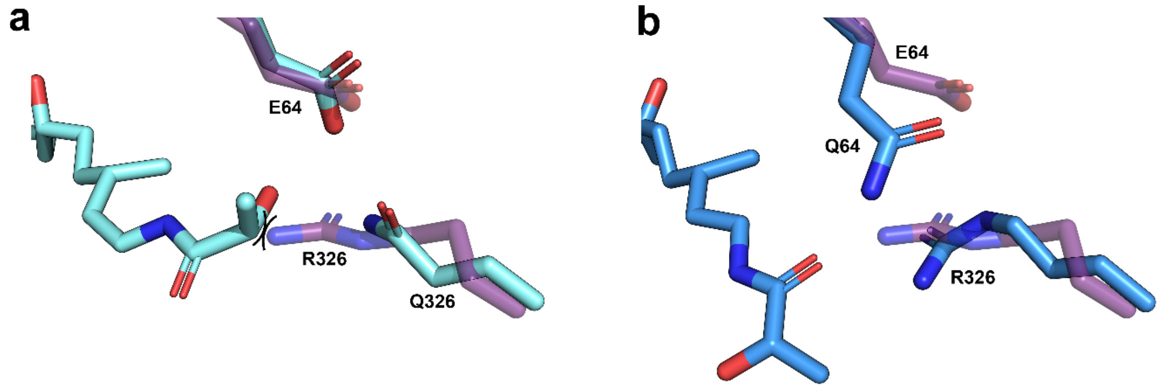

Supplementary Figure 13 │ Analysis of LC-KA05 binding into the LkcE active site. (**a**) Superposition of the R326Q/LC-KA05 (**1**) structure (cyan) and that of the *holo* wild type (mauve) shows that if **1** bound into the wild type in the same orientation as in the R326Q/LC-KA05 complex, its C-24 hydroxyl would sterically clash with R326. (**b**) Superposition of the E64Q/LC-KA05 structure (blue) and that of the *holo* wild type (mauve). In the E64Q structure, the substrate adopts an orientation such that there is no longer a steric clash with R326.

Supplementary Tables

Supplementary Table 1 │ Oligonucleotide primers used in this study. Mutated sites are in bold.

Name	Sequence, 5′-3′ direction
Primers used for lkcE *deletion plasmid*	
LkcE deletion_F	GCAGGCCGAGCCCAGGAAGTGATGACCGTGGTAGAAGCAATTCCGGGGATCCGTCGACC
LkcE deletion_R	GACAGGCGGGCGGGTGCTGGCTCACACGCCCATGAATTCTGTAGGCTGGAGCTGCTTC
Primers used to verify lkcE *deletion*	
PCR conf_F	TGCGGTGGTGGCAGACCGTG
PCR conf_R	GCGGCGAGGTCGGTGTATCG
PCR conf_I	ACTCGCCTGCAAGCCCGGTC
Primers used for site-directed mutagenesis in the lkcE *gene*	
E64A_F	GTCGACATGGGCGTG**GCA**CACTTCAACGAGAAG
E64A_R	CTTCTCGTTGAAGTG**TGC**CACGCCCATGTCGAC
E64Q_F	GTCGACATGGGCGTG**CAA**CACTTCAACGAGAAG
E64Q_R	CTTCTCGTTGAAGTG**TTG**CACGCCCATGTCGAC
Y182F_F	GACTACAACCTCATG**TTC**GTCGCGCTGTCGTT
Y182F_R	ACGACAGCGCGAC**GAA**CATGAGGTTGTAGTC
R326L_F	ATGGGCAGCCTGACC**CTC**AACTGCAACGTGCTC
R326L_R	GAGCACGTTGCAGTT**GAG**GGTCAGGCTGCCCAT
R326Q_F	ATGGGCAGCCTGACC**CAG**AACTGCAACGTGCTC
R326Q_R	GAGCACGTTGCAGTT**CTG**GGTCAGGCTGCCCAT

**Supplementary Table 2 │ List and description of vectors used in this study.**			
Name	Description	Application	Reference
pBG102	Derivative of pET27	Used to generate N-terminal translational fusions with His_6 and SUMO tags	Center for Structural Biology, Vanderbilt University
pBG102-LkcE	pBG102 containing the gene encoding LkcE	Production of recombinant LkcE	This study
pBG102-LkcE_E64A	Derived from pBG102-LkcE; LkcE has Glu64Ala mutation	Evaluation of the role of residue Glu64	This study
pBG102-LkcE_E64Q	Derived from pBG102-LkcE; LkcE has Glu64Gln mutation	Evaluation of the role of residue Glu64	This study
pBG102-LkcE_Y182F	Derived from pBG102-LkcE; LkcE has Tyr182Phe mutation	Evaluation of the role of residue Tyr182	This study
pBG102-LkcE_R326L	Derived from pBG102-LkcE; LkcE has Arg326Leu mutation	Evaluation of the role of residue Arg326	This study
pBG102-LkcE_R326Q	Derived from pBG102-LkcE; LkcE has Arg326Gln mutation	Evaluation of the role of residue Arg326	This study
pIJ773	Template plasmid containing the apramycin resistance gene *aac(3)IV* and the *oriT* of plasmid RP4	Used to generate the *lkcE* inactivation construct	1
Cosmid Lc2B12	Derivative of SuperCos1 containing a portion of the lankacidin gene cluster	Used to inactive *lkcE* in *S. rochei*	2
pIJ790	Temperature sensitive λRed recombination helper plasmid	λRED recombination plasmid	1

Supplementary Table 3 │Data collection and refinement statistics on LkcE crystal structures.

	SeLkcE	$LkcE_{WT}$	R326Q	E64Q
**Data collection**				
Space group	$P4_12_12$	$P4_12_12$	$P4_12_12$	$P4_12_12$
Cell dimensions				
a, *b*, *c* (Å)	125.30, 125.30, 154.53	125.50, 125.50; 156.69	125.55, 125.55; 156.66	124.83, 124,83, 157.39
α, β, γ (°)	90.00, 90.00, 90.00	90.00, 90.00, 90.00	90.00, 90.00, 90.00	90.00, 90.00, 90.00
Resolution (Å)	48.66-3.15* (3.2-3.15)	48.98-2.80* (3.00-2.80)**	48.99-2.50* (2.60-2.50)**	48.90-3.03* (3.11-3.03)**
R_{merge}	0.131 (0.802)	0.05 (0.56)	0.05 (0.64)	0.11 (0.76)
$I / \sigma I$	13.62 (2.58)	22.3 (3.8)	20.24 (2.42)	30.69 (5.67)
CC(1/2)	1.00 (0.78)	0.99 (0.89)	0.99 (0.75)	1.00 (0.95)
Completeness (%)	99.9 (99.3)	99.9 (100.0)	99.6 (99.4)	99.8 (97.2)
Redundancy	7.6 (7.0)	7.4 (7.5)	4.4 (4.4)	29.7 (27.9)
**Refinement**				
Resolution (Å)	48.66-3.15	48,98-2.80	48.99-2.50	48.90-3.03
No. reflections	21897	31467	41613	23588
R_{work} / R_{free}	0.198 / 0.240	0.237 / 0.256	0.206 / 0.244	0.191 / 0.247
No. atoms				
Protein	6807	6835	6888	6907
Ligand/ion	112	195	129	79
Water	216	181	230	136
B-factors				
Protein[§]	70.52	87.81	61.75	70.26
Ligand/ion	67.96	77.40	58.16	77.01
Water	56.01	107.13	73.00	52.88
R.m.s. deviations				
Bond lengths (Å)	0.010	0.008	0.015	0.012
Bond angles (°)	1.13	1.10	1.79	1.61

*A single crystal was used for each data set; **Values in parentheses are for highest-resolution shell.
[§] The corresponding B-factors for the protein from the Wilson plots are: 75.3, 82.2, 60.8 and 72.9, respectively. There is good agreement between these two sets of B-factors, indicative of significant dynamic disorder at the atomic level in the structures.

Supplementary Table 4 │ Analytical data obtained by HPLC-MS on compounds investigated in this study.

Compound	Calc. *m/z* (Da)	Number of peaks	Retention time(s) (min)[a]	Characteristic MS peaks (ionization mode)
**1** LC-KA05	505.27	2	Culture extracts: 21.8, 22.9 (Fig. S7)<br>In vitro: 23.4 (Fig. S10) and 24.5 (Fig. S11)	544 ($[M+K]^+$), 528 ($[M+Na]^+$), 506 ($[M+H]^+$), 446, 428, 339 (ESI positive)
**2** Lankacidinol A	503.25	3–5	Culture extracts: 21.8, 22.6, 23.0 (Fig. S7)<br>In vitro: 20.7, 22.5, 23.3, 26.1, 26.7 (Fig. S11)	542 ($[M+K]^+$), 526 ($[M+Na]^+$), 504 ($[M+H]^+$), 486, 426, 354, 337 (ESI positive)
Lankacidin A	501.24	1	Culture extracts: 28.7 (Fig. S7)	524 ($[M+Na]^+$), 502 ($[M+H]^+$), 484, 424, 354, 337 (ESI positive)
**3** Lankacidin C	459.22	1	Culture extracts: 25.6 (Fig. S7)	460 ($[M+H]^+$), 400 (ESI positive)
**6** Hydrolytic (deacetylated) derivative of LC-KA05	463.26	1 or 2	Culture extracts: 12.9, 13.9<br>In vitro: 15.2<br>(Both in Fig. S9)	462 ($[M–H]^–$) (ESI negative)<br>502 ($[M+K]^+$), 486 ($[M+Na]^+$), 464 ($M+H]^+$), 446, 428, 339 (ESI positive)
**7** Eliminated derivative of LC-KA05	445.25	1	Culture extracts: 25.2<br>In vitro: 26.0<br>(Both in Fig. S9)	484 ($[M+K]^+$), 468 ($[M+Na]^+$), 446 ($[M+H]^+$), 428, 339 (ESI positive)
**8** Cyclized 7	443.23	2	In vitro: 27.6, 28.0 (Fig. S9)	482 ($[M+K]^+$), 466 ($[M+Na]^+$), 444 ($[M+H]^+$), 426, 355, 337 (ESI positive)
**9** Lankacidinol C (cyclized 6)	461.24	2	In vitro: 17.5, 18.7 (Fig. S9)	498 ($[M–2H+K]^–$), 482 ($[M–2H+Na]^–$), 460 ($[M–H]^–$), 384 (ESI negative)

[a]Analysis of several compounds (**1**, **2**, **6**, **8** and **9**) gave multiple peaks, which we attribute to the presence of isomerization in the two conjugated systems (C8–C11 and C14–C17). In addition, for compounds **1**, **2, 6** and **7**, we noted differences both in retention times and the number of peaks when analysis was carried out on culture extracts vs. substrates/products of in vitro assays. We can cite several potential reasons for these variations. Although all of the analyses were performed under the same conditions (column, elution profile, etc.), the two-year period over which they were carried out may have seen some degradation of the column material. In addition, differential exposure to light between the extracts and the in vitro assays may have led to differing extents of isomerization in the conjugated systems. We could nonetheless make confident peak assignments based on the mass spectra and comparison to the control analyses (extracts of the lkcE mutant vs. the wild type, analysis of active LkcE in vitro vs. inactivated protein).

Supplementary References

1. Gust, B., Challis, G. L., Fowler, K., Kieser, T. & Chater, K. F. *Proc. Natl. Acad. Sci. U. S. A.* **100**, 1541–1546 (2003).
2. Dickschat, J. S. *et al. ChemBioChem* **12**, 2408–2412 (2011).
3. Arakawa, K., Sugino, F., Kodama, K., Ishii, T. & Kinashi, H. *Chem. Biol.* **12**, 249–256 (2005).
4. Altschul, S. F., Gish, W., Miller, W., Myers, E. W. & Lipman, D. J. *J. Mol. Biol.* **215**, 403–410 (1990).
5. Ng, B-G., Han, J.-W., Lee, D. W., Choi, G. J., Kim, B. S. *J. Antibiot.* (2018). doi.org/10.1038/s41429-017-0023-x.
6. Kachalova, G., Decker, K., Holt, A., & Bartunik, H. D. *Proc. Natl. Acad. Sci. U. S. A*. **108**, 4800–4805 (2011).
7. Binda, C., Newton-Vinson, P., Hubálek, F., Edmondson, D. E. & Mattevi, A. *Nat. Struct. Biol.* **9**, 22–26 (2002).
